# Phosphorylation of Calcineurin at a Novel Serine-Proline Rich Region Orchestrates Hyphal Growth and Virulence in *Aspergillus fumigatus*

**DOI:** 10.1371/journal.ppat.1003564

**Published:** 2013-08-22

**Authors:** Praveen R. Juvvadi, Christopher Gehrke, Jarrod R. Fortwendel, Frédéric Lamoth, Erik J. Soderblom, Erik C. Cook, Michael A. Hast, Yohannes G. Asfaw, M. Arthur Moseley, Trevor P. Creamer, William J. Steinbach

**Affiliations:** 1 Department of Pediatrics, Division of Pediatric Infectious Diseases, Duke University Medical Center, Durham, North Carolina, United States of America; 2 Department of Molecular Genetics and Microbiology, Duke University Medical Center, Durham, North Carolina, United States of America; 3 Duke Proteomics Facility, Institute for Genome Sciences and Policy, Duke University, Durham, North Carolina, United States of America; 4 Department of Molecular and Cellular Biochemistry and Center for Structural Biology, University of Kentucky, Lexington, Kentucky, United States of America; 5 Department of Biochemistry, Duke University Medical Center, Durham, North Carolina, United States of America; 6 Division of Laboratory Animal Resources, Duke University Medical Center, Durham, North Carolina, United States of America; University of Toronto, Canada

## Abstract

The fungus *Aspergillus fumigatus* is a leading infectious killer in immunocompromised patients. Calcineurin, a calmodulin (CaM)-dependent protein phosphatase comprised of calcineurin A (CnaA) and calcineurin B (CnaB) subunits, localizes at the hyphal tips and septa to direct *A. fumigatus* invasion and virulence. Here we identified a novel serine-proline rich region (SPRR) located between two conserved CnaA domains, the CnaB-binding helix and the CaM-binding domain, that is evolutionarily conserved and unique to filamentous fungi and also completely absent in human calcineurin. Phosphopeptide enrichment and tandem mass spectrometry revealed the phosphorylation of *A. fumigatus* CnaA *in vivo* at four clustered serine residues (S406, S408, S410 and S413) in the SPRR. Mutation of the SPRR serine residues to block phosphorylation led to significant hyphal growth and virulence defects, indicating the requirement of calcineurin phosphorylation at the SPRR for its activity and function. Complementation analyses of the *A. fumigatus* Δ*cnaA* strain with *cnaA* homologs from the pathogenic basidiomycete *Cryptococcus neoformans*, the pathogenic zygomycete *Mucor circinelloides*, the closely related filamentous fungi *Neurospora crassa*, and the plant pathogen *Magnaporthe grisea*, revealed filamentous fungal-specific phosphorylation of CnaA in the SPRR and SPRR homology-dependent restoration of hyphal growth. Surprisingly, circular dichroism studies revealed that, despite proximity to the CaM-binding domain of CnaA, phosphorylation of the SPRR does not alter protein folding following CaM binding. Furthermore, mutational analyses in the catalytic domain, CnaB-binding helix, and the CaM-binding domains revealed that while the conserved PxIxIT substrate binding motif in CnaA is indispensable for septal localization, CaM is required for its function at the hyphal septum but not for septal localization. We defined an evolutionarily conserved novel mode of calcineurin regulation by phosphorylation in filamentous fungi in a region absent in humans. These findings suggest the possibility of harnessing this unique SPRR for innovative antifungal drug design to combat invasive aspergillosis.

## Author Summary

Invasive fungal infections are a leading cause of death in immunocompromised patients. Translating molecular understanding into tangible clinical benefit has been difficult due to the fact that fungal pathogens and their hosts have similar physiology. The calcineurin pathway is an important signaling cascade in all eukaryotes, and calcineurin inhibitors are powerful immunosuppressants that have revolutionized medicine. Through both genetic and pharmacologic inhibition, we have established that calcineurin is vital for invasive fungal disease. Although the currently available calcineurin inhibitors are active *in vitro* against the major invasive fungal pathogens, they are also immunosuppressive in the host, limiting therapeutic effectiveness. Here we defined an evolutionarily conserved novel mode of calcineurin regulation by phosphorylation in filamentous fungi that is responsible for virulence in the opportunistic human pathogen, *Aspergillus fumigatus*. This phosphorylation occurs on a cluster of four serine residues located in a unique serine-proline rich domain of calcineurin that is absent in humans. This finding of a new fungal-specific mechanism controlling hyphal growth and virulence represents a new potential target for antifungal drug therapy.

## Introduction

Invasive fungal infections are a leading cause of death in immunocompromised patients [1]. With a 40–60% mortality rate, invasive aspergillosis, caused by the filamentous fungus *Aspergillus fumigatus*, is the most frequent fungal cause of mortality [2]. Through both genetic and pharmacologic inhibition, we have established that the conserved phosphatase calcineurin is necessary for invasive fungal disease [3], [4]. Although currently available calcineurin inhibitors FK506 and cyclosporine A are active *in vitro* against *A. fumigatus* [5], they are also immunosuppressive in the host, limiting therapeutic effectiveness. Our goal is to translate fungal biology into tangible clinical benefit by identifying targets that specifically inhibit fungal calcineurin, resulting in fungal killing without suppressing the immune system of the host.

Calcineurin is a Ca^2+^/calmodulin (CaM)-dependent protein phosphatase comprised of a catalytic A and regulatory B subunit heterodimeric complex [6]. Calcineurin is activated after Ca^2+^/CaM binds to calcineurin A at the CaM-binding domain (CaMBD), adjacent to the calcineurin B binding helix (CnBBH) in its regulatory domain and displaces the auto-inhibitory domain (AID) [6], [7].

Although calcineurin is conserved from yeasts to human, it exhibits diverse roles in different cell types, evidenced by modulating immune responses, impacting muscle development, neuronal plasticity and cell death in mammalian cells [8]–[11], and influencing cation homeostasis, morphogenesis, cell wall integrity, mating, and stress responses in yeasts [12]–[15]. In the fission yeast *Schizosaccharomyces pombe*, calcineurin participates in morphogenesis by affecting septal positioning, spindle body organization, and membrane trafficking [16], [17]. In the pathogenic yeasts *Candida albicans* and *Cryptococcus neoformans*, calcineurin regulates growth at alkaline pH and elevated temperature, membrane stress, and virulence [18]–[20]. In filamentous fungi, calcineurin is important for cell cycle progression, hyphal branching, stress adaptation, sclerotial development and formation of the infectious appressorium in a plant pathogen [21]–[25].

As a protein phosphatase, calcineurin is known to dephosphorylate specific substrates [26]. However, few reports have focused on phosphorylation of calcineurin as a mechanism of its own activation. King and Huang [27] first reported that bovine brain calcineurin contains sub-stoichiometric amounts of covalently bound phosphate, suggesting calcineurin regulation by phosphorylation. While bovine calcineurin phosphorylation by CK1 yielded no change in resultant activity [28], its phosphorylation by CaM Kinase II and PKC in the CaMBD (S411) inactivated it and decreased its affinity for substrates [29]–[31]. Although this phosphorylation was inhibited upon CaM binding [29], it did not significantly alter the binding of CaM [32]. Recently, calcineurin from *S. pombe* was shown to be activated after phosphorylation by the check point kinase Cds1 at the similarly positioned serine residue within the CaMBD (S459), and at another site at the C-terminus (S521) [33].

We and others have previously determined that calcineurin is required for hyphal growth and virulence of *A. fumigatus* [3], [34]. We subsequently showed that the calcineurin complex (CnaA and CnaB) localizes at both the hyphal tips and septa to direct proper hyphal growth and regular septum formation, and that the regulatory subunit (CnaB) is essential for activation of the catalytic subunit (CnaA) *in vivo* [35], [36].

Here we performed mutational analyses in the functional domains of *A. fumigatus* CnaA to investigate those required for hyphal growth, CnaA septal localization, phosphatase function, and virulence. We uncovered six novel findings, including (i) the linker between the CnBBH and CaMBD, contains a region unique to filamentous fungi (completely absent in humans), that is rich in serine and proline residues (404-PTSVSPSAPSPPLP-417; designated “SPRR” for Serine Proline Rich Region) and is phosphorylated *in vivo* at all 4 clustered serine residues (S406, S408, S410 and S413), (ii) complementation of the *A. fumigatus* Δ*cnaA* mutant strain with calcineurin A homologs from other fungi defined a filamentous fungal-specific phosphorylation of the SPRR in CnaA, suggesting its evolutionarily conserved importance in fungal hyphal growth, (iii) GSK-3β, CK1, CDK1 and MAP kinase as potential kinases that phosphorylate the SPRR, implicating their role in the regulation of *A. fumigatus* CnaA, (iv) mutations in the SPRR did not affect septal localization of CnaA but resulted in significant hyphal growth and virulence defects, implicating the importance of calcineurin phosphorylation for its function in *A. fumigatus* and its possibility as a new antifungal target, (v) CaM is not required for septal localization of CnaA but is required for its function at the hyphal septum, and (vi) the PxIxIT substrate binding motif in CnaA is required for its localization at the hyphal septum.

## Results

### Truncations of *A. fumigatus* CnaA revealed important domains required for its function and septal localization

**Figure 1:**
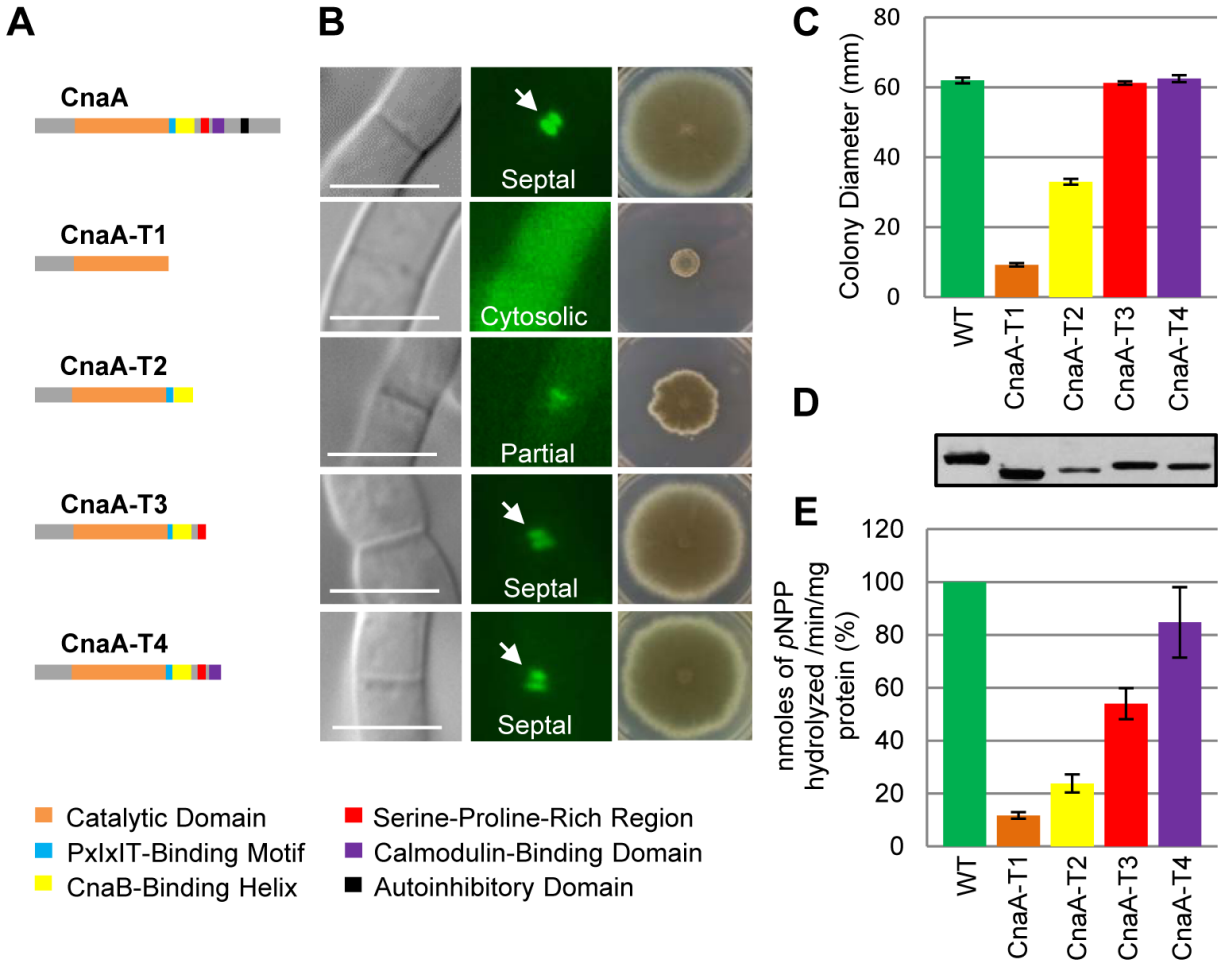
Truncations of *A. fumigatus* CnaA revealed important domains required for growth and septal localization. (**A**) Scheme of *cnaA* truncations and domain organization. Constructs were expressed with native *cnaA* promoter and *egfp* tag at the C-terminus to visualize localization and complementation in the Δ*cnaA* mutant. Full length CnaA (1–559 aa), CnaA-T1 (1–347 aa including the catalytic domain), CnaA-T2 (1–400 aa including the CnaB-Binding Helix), CnaA-T3 (1–424 aa including the SPRR linker), and CnaA-T4 (1–458 aa including the Ca^2+^/Calmodulin-Binding Domain) are shown. (**B**) CnaA localization after 24 h growth is indicated as cytoplasmic, partial, or septal. Radial growth was assessed by inoculating 1×10^4^ conidia on GMM agar after 5 days at 37°C. (**C**) Radial growth is depicted as mean diameter after 5 days growth in triplicate. (**D**) Western detection of CnaA-EGFP fusion proteins using anti-GFP polyclonal antibody and peroxidase labeled anti-rabbit IgG secondary antibody. (**E**) Calcineurin activity was determined using *p*-nitrophenyl phosphate as a substrate. Data from two separate experiments, each with 6 technical replicates are presented as mean ± SD of nanomoles of *p*NPP released/min/mg protein.

To characterize domains required for CnaA activity and septal localization, we generated *A. fumigatus* strains expressing a series of truncated *cnaA* cDNAs (*cnaA-T1*, *cnaA-T2*, *cnaA-T3* and *cnaA-T4*) under the control of its native promoter in the Δ*cnaA* mutant strain (Figure 1A). While the expression of *cnaA-T1*, containing only the catalytic domain (1–347 aa), did not complement the hyphal growth defect of the Δ*cnaA* mutant strain and mislocalized CnaA in the cytoplasm (Figure 1B and 1C), expression of *cnaA-T2* that included the CnBBH region (1–400 aa) showed partial growth recovery, indicating that this fragment may bind to CnaB *in vivo* and partially function by less efficiently localizing at the septum (Figure 1B and 1C). However, expression of *cnaA-T3* (1–425 aa), containing the linker region spanning 23 aa between the CnBBH and CaMBD (Figure 1A; indicated in red), completely restored hyphal growth and efficiently localized CnaA at the septum (Figure 1B and 1C). This indicated that the CaMBD and AID are not required for septal targeting of CnaA. Complete hyphal growth recovery observed in the CnaA-T3 strain also suggested the possibility of a constitutively active calcineurin due to the absence of the AID. Expression of *cnaA-T4*, including the CaMBD but not the AID (1–458 aa), also completely restored hyphal growth and properly localized CnaA at the septa (Figure 1B and 1C). Expression of all the constructs was confirmed by Western analysis (Figure 1D).

**Figure 2:**
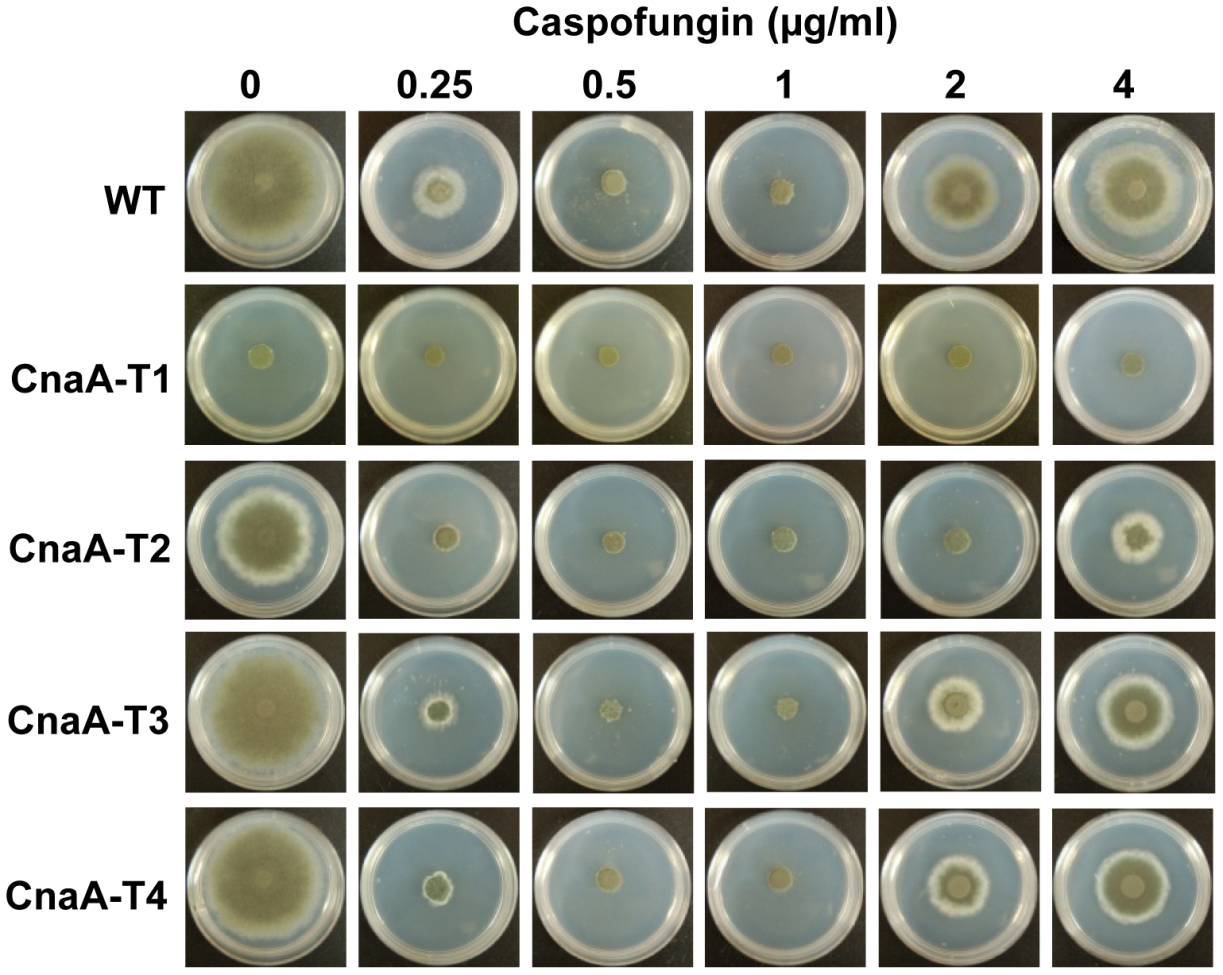
Sensitivity of the CnaA truncation strains to Caspofungin. CnaA truncation expression strains were cultured on GMM agar in the presence of varying concentrations of the cell wall inhibitor caspofungin for 5 days. The CnaA-T1 strain, which lacks all the functional domains of CnaA, exhibits a severe growth defect and lacks caspofungin-mediated paradoxical growth due to a lack of calcineurin activity. In comparison to the WT the CnaA-T3 and CnaA-T4 strains show more sensitivity to 0.25 µg/ml caspofungin but recovery of paradoxical growth at 2 and 4 µg/ml caspofungin. The CnaA-T2 strain, without the SPRR, shows paradoxical growth only at 4 µg/ml caspofungin.

Our previous studies have shown that the “paradoxical effect” (attenuation of the antifungal activity of the echinocandin drug caspofungin at elevated concentrations) is calcineurin-mediated, and that paradoxical growth is abolished in the Δ*cnaA* mutant strain lacking calcineurin activity [37]. While the CnaA-T1 strain showed no paradoxical growth (Figure 2), the CnaA-T2 strain exhibited partial recovery of paradoxical growth only at 4 µg/ml of caspofungin. In comparison to the wild-type, the CnaA-T3 and CnaA-T4 strains displayed more sensitivity to 0.25 µg/ml caspofungin, indicating less calcineurin activity, but showed almost wild-type equivalent paradoxical growth recovery at 4 µg/ml caspofungin. Concordant with these findings, the CnaA-T1 and CnaA-T2 strains (Figure 1E) showed a significant reduction in calcineurin activity (86% and 80%, respectively), and the CnaA-T3 strain showed only a 28% decrease in activity. Inclusion of the CaMBD in the CnaA-T4 strain restored wild-type level of calcineurin activity (Figure 1E). The growth restoration of the CnaA-T3 and CnaA-T4 strains may also be attributed to constitutively active calcineurin due to the truncation of the C-terminal autoinhibitory domain. Taken together, these results indicated that the major determinants/residues for hyphal growth restoration in the CnaA-T3 and CnaA-T4 strains and CnaA septal targeting may be present in this newly described linker region between the CnBBH and the CaMBD of CnaA. However, it is possible that targeting CnaA to the hyphal septum occurs either independently or by binding of the linker region to other unknown protein(s).

### Identification of a unique Serine-Proline Rich Region (SPRR) in the linker between the CnaB-binding helix and the Ca^2+^/calmodulin binding domain of CnaA

**Figure 3:**
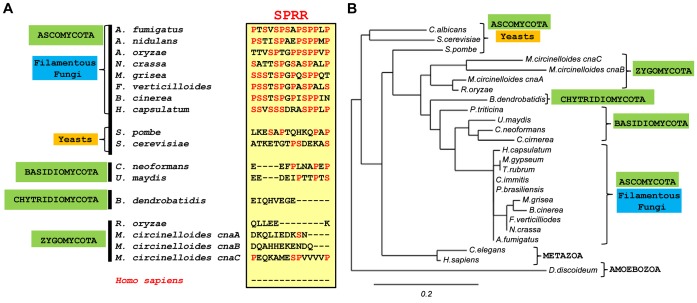
Conservation of a unique Serine-Proline Rich Region in filamentous fungi. (**A**) Alignment of the SPRR among filamentous ascomycete fungi, yeasts, other fungi, and human. Conservation of serine and proline residues within the SPRR among filamentous fungi suggests their importance for hyphal growth; hyphal growth appears related to the potential ability for phosphorylation in the SPRR domain (box). Importantly, there is no homology in humans. (**B**) Phylogenic analysis of the CnaA region including the CnBBH, linker domain with the SPRR, and the CaMBD performed on the Phylogeny.fr platform. Sequences aligned with MUSCLE (v3.7) configured for highest accuracy. Phylogenetic tree was reconstructed using the maximum likelihood method implemented in the PhyML program (v3.0 aLRT) and graphically represented with TreeDyn (v198.3).

Because the truncated forms of CnaA revealed the importance of the linker region between the CnBBH and the CaMBD for hyphal growth and septal localization of CnaA, we performed multiple sequence alignments of *A. fumigatus* CnaA with homologs from other organisms. This alignment confirmed a high degree of conservation within the catalytic domain, CnBBH, and the CaMBD across different species (data not shown). However, the linker region showed marked variation in different species (Figure 3A and S1). There are no structural data available on fungal calcineurins, so we modeled the *A. fumigatus* CnaA-CnaB complex based on the available human calcineurin complex structure [38]. Our calcineurin modeling attempts did not reveal any known structure in this linker region, as it seemed to be highly disordered. Inside the 23-residue *A. fumigatus* linker region there is a specific 14 residue domain (404-PTSVSPSAPSPPLP-417) that is relatively conserved in filamentous fungi and completely absent in the human calcineurin α-catalytic subunit (Figure 3A). We designated this as the “Serine-Proline Rich Region” (SPRR), based on the preponderance of serine and proline residues. A phylogenetic tree constructed from an alignment of the CnBBH-linker-CaMBD domains from diverse organisms showed that, although the CnBBH and CaMBD were nearly identical for all species, the linker, and specifically the SPRR, clearly distinguished the filamentous fungi from other species (Figure 3B). This suggested that the SPRR could be evolutionarily important for filamentous hyphal growth.

### The CnaA Serine-Proline Rich Region is conserved among filamentous fungi and is important for hyphal growth

**Figure 4:**
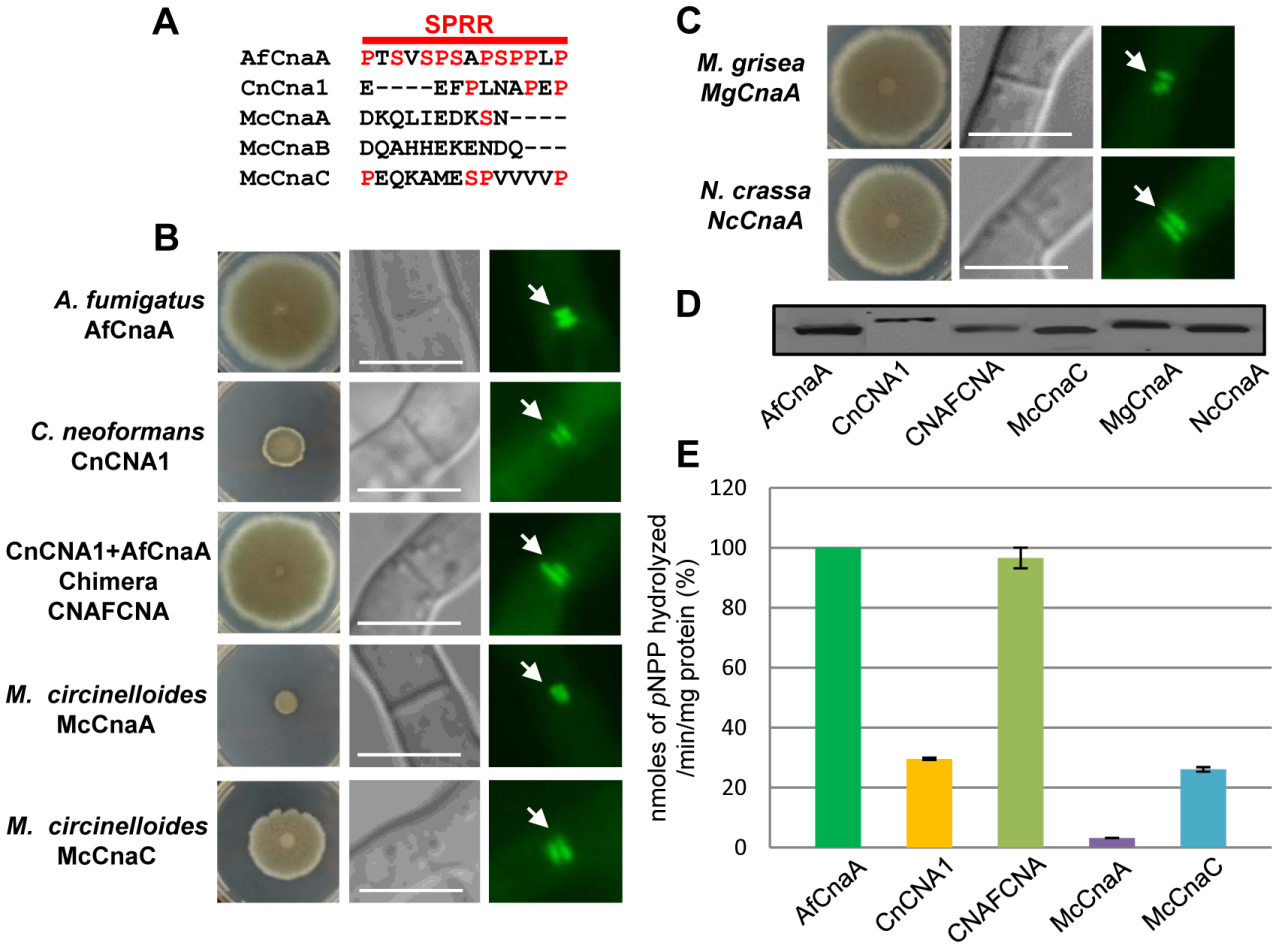
The conserved CnaA Serine-Proline Rich Region is important for hyphal growth. (**A**) Alignment of *C. neoformans* CnCna1 and the three calcineurin A catalytic subunit proteins (McCnaA, McCnaB and McCnaC) in *M. circinelloides* with *A. fumigatus* CnaA SPRR (AfCnaA). (**B**) Complementation of the *A. fumigatus* Δ*cnaA* mutant with native AfCnaA, *C. neoformans* CnCNA1, a CNAFCNA chimera consisting of the N-terminus catalytic domain of *C. neoformans CNA1* and the C-terminal portion of *AfcnaA* consisting of the CnBBH, SPRR, CaMBD and the AID, *M. circinelloides* McCnaA, and MccnaC. (**C**) Complementation of the *A. fumigatus* Δ*cnaA* mutant with *M. grisea MgcnaA* and *N. crassa NccnaA*. Strains were assessed for growth complementation and CnaA septal localization (arrow) by fluorescence microscopy. (**D**) Western detection of the calcineurin A proteins expressed in the complemented strains. (**E**) Calcineurin activity after 24 h growth using *p*-nitrophenyl phosphate as a substrate. Data from two separate experiments, each with 6 technical replicates are presented as mean ± SD of nanomoles of *p*NPP released/min/mg protein.

The *A. fumigatus* SPRR has little homology with the region in the yeast *S. cerevisiae*, and, importantly, the region is absent in human calcineurin. We transformed the *A. fumigatus* Δ*cnaA* mutant strain with CnaA homologs from human (CnAα) and *S. cerevisiae* (*CNA1*) and found no hyphal growth recovery and cytosolic CnaA localization (data not shown). We then complemented our *A. fumigatus* Δ*cnaA* mutant strain with CnaA counterparts from other phylogenetically distinct fungi belonging to the phyla basidiomycota and zygomycota (Figure 4A and 4B). *CNA1* from *C. neoformans* minimally restored the hyphal growth defect (Figure 4B) but septal localization was seen, indicating that *C. neoformans* CNA1 contained the determinants required for septal localization but not hyphal growth. Western analysis confirmed the expression of *C. neoformans CNA1* (Figure 4D). Next, we constructed a chimera (CNAFCNA) consisting of the N-terminal *C. neoformans CNA1* catalytic domain and the C-terminal regulatory domain of *A. fumigatus cnaA*, including the SPRR (Figure 4B). This chimera completely restored hyphal growth, indicating that the SPRR is important for regulating hyphal growth.

We also complemented the *A. fumigatus* Δ*cnaA* mutant strain with calcineurins from a phylogenetically unrelated zygomycete fungus, *Mucor circinelloides*, which grows as extended hyphae but lacks septation (Figure 4B). *M. circinelloides* has three calcineurin A homologs (designated as *MccnaA*, *MccnaB* and *MccnaC*; Lee SC et al, communicated) and we utilized the genes encoding MccnaA and MccnaC that showed variability in the SPRR (Figure 4A). Although both McCnaA and MccnaC localized to the septum, MccnaC expression showed greater recovery of the hyphal growth defect (Figure 4B). Clustal alignment with the *A. fumigatus* CnaA SPRR revealed that MccnaC contained 3 prolines and a serine residue, but MccnaA had only a single serine residue (Figure 4A), suggesting that the partial growth complementation observed with MccnaC may be due to partial homology to the *A. fumigatus* SPRR. To confirm this, we transformed the *A. fumigatus* Δ*cnaA* mutant strain with *cnaA* homologs from closely related filamentous fungi *Magnaporthe grisea* and *Neurospora crassa* that have greater homology in the SPRR (Figure 3A and 4C). Both *M. grisea* and *N. crassa* calcineurins fully complemented the growth defect, restored calcineurin activity (data not shown) and also localized to the hyphal septa (Figure 4C). Western analysis confirmed the expression of the respective calcineurins from *M. circinelloides*, *M. grisea*, and *N. crassa* (Figure 4D). While calcineurin activity was decreased by ∼70% in the *C. neoformans CNA1* complemented strain, concomitant with the decreased radial growth, expression of the chimera (CNAFCNA), which contained the SPRR, completely restored both calcineurin activity and hyphal growth (Figure 4E). Although the McCnaC partially complemented the hyphal growth defect, that replacement strain possessed less calcineurin activity. Taken together, these results indicated that SPRR is important for regulating proper hyphal growth, calcineurin activity, and CnaA septal localization.

### CnaA is phosphorylated in the Serine-Proline Rich Region

**Figure 5:**
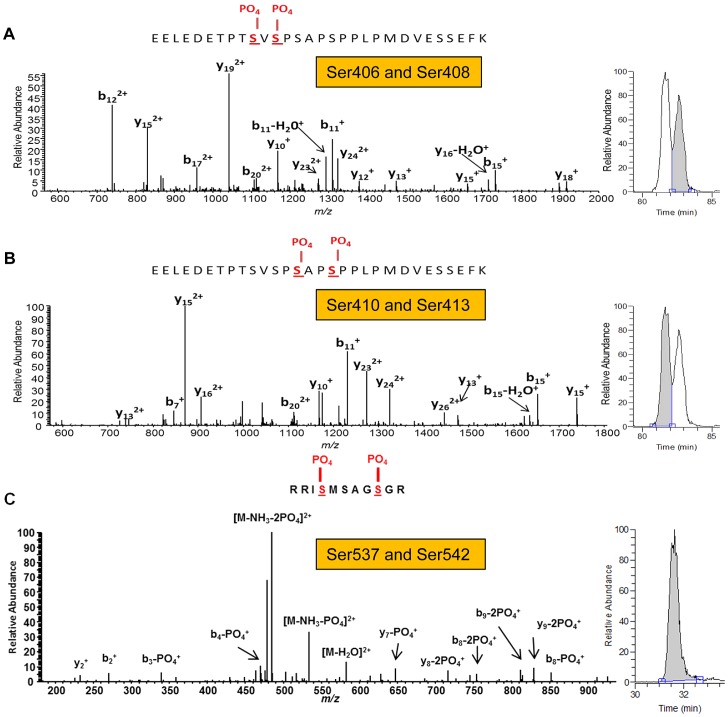
CnaA is phosphorylated at the Serine-Proline Rich Region *in vivo*. (**A, B**) Tandem mass spectra of (A) EELEDETPT[pS]V[pS]PSAPSPPLPMDVESSEFK and (B) EELEDETPTSVSP[pS]AP[pS]PPLPMDVESSEFK from CnaA subunit reveal four unique phosphorylated serine residues (406, 408, 410 and 413) in close proximity. The presence of each identified C-terminal (y) and N-terminal (b) product ions are indicated within the peptide sequence. For additional verification of the unique localization of phosphorylation between the two peptides, corresponding full MS extracted ion chromatograms of *m/z* 1131.1415 (+/−10 ppm) are shown on the right of each mass spectrum and illustrate a clear chromatographic shift in retention time between the species. (**C**) Tandem mass spectrum of RI[pS]MSAGSGR from CnaA subunit revealed two unique phosphorylated serine residues (537 and 542). The presence of each identified C-terminal (y) and N-terminal (b) product ions are indicated within the peptide sequence. The single peak in the corresponding full MS extracted ion chromatogram of *m/z* 591.2272 (+/−10 ppm) shown to the right of the mass spectrum indicates that S537 and S542 were the only two phosphorylated residues within the peptide.

The concentration of serine and proline residues in SPRR may create a hydrophobic environment, and the PPLP-motif, predicted to be a WW-domain protein binding motif, may contribute to protein-protein interactions [39]. Phosphorylation at serine or threonine residues that precede a proline, referred to as proline-directed phosphorylation, is known to play an essential role in the regulation of cellular processes such as cell proliferation and differentiation [40]. The two proline residues P409 and P414, preceded by serine residues at positions 408 and 413, respectively, prompted us to examine the phosphorylation of *A. fumigatus* CnaA. We found that the isolated *A. fumigatus* CnaA-EGFP fusion protein reacted with the anti-phosphoserine antibody, indicating that CnaA was phosphorylated *in vivo* (data not shown). Further phosphoproteomic analyses by LC-MS/MS identified 6 serine residues phosphorylated in *A. fumigatus* CnaA (Figure 5), including all four serine residues clustered in the SPRR at positions 406, 408, 410 and 413 (Figure 5A and 5B), and two additional serine residues in the C-terminus at positions 537 and 542 (Figure 5C). Validation of phosphorylation site localization was performed using the AScore algorithm (Table 1). Furthermore, we also identified that the calcineurin regulatory subunit, CnaB, which was co-purified with CnaA, was also phosphorylated at two serine residues (Ser21 and Ser33) at its N-terminus (Figure S2 and Table 1).

**Table 1:** Phosphorylation sites within CnaA and CnaB subunits identified by TiO_2_ enrichment and LC-MS/MS analysis.

Protein	Peptide Sequence^a^	Phosphorylated Residue	*m/z*	Charge	Mass Error (ppm)	Mascot Ion Score^b^	Ascore Localization Probability^c^
CnaA	EELEDETPTSV[**pS**]PSAPSPPLPMDVESSEFK	S408	1104.4843	3	−1.6	46	99%
CnaA	EELEDETPT[**pS**]V[**pS**]PSAPSPPLPMDVESSEFK	S406, S408	1131.1415	3	−2.1	54	80%, 80%
CnaA	EELEDETPTSVSP[**pS**]AP[**pS**]PPLPMDVESSEFK	S410, S413	1131.1415	3	−0.5	75	90%, 99%
CnaA	EELEDETPT[**pS**]VSPSAPSPPLPMDVESSEFKR	S406	1156.5159	3	−3.4	94	90%
CnaA	RI[**pS**]MSAGSGR	S537	551.2449	2	−4.2	72	99%
CnaA	RI[**pS**]MSAG[**pS**]GR	S537, S542	591.2272	2	−5.3	66	99%, 99%
CnaB	RA[**pS**]VGTSQLLDNIV[**pS**]ASNFDRDEVDR	S21, S33	1008.7813	3	−5.5	94	99%, 99%
CnaB	RA[**pS**]VGTSQLLDNIVSASNFDRDEVDR	S21	982.1329	3	1.5	106	99%

a [pS] indicates phosphorylated residue.

b Mascot identity score of >41 indicates identity or extensive homology (p<0.05).

c Probability of phosphorylated residue localization based on Ascore algorithm.

CnaA subunit and CnaB subunit peptides contained uniquely identified phosphorylation residues or combinations of phosphorylation residues. Due to the proximity of multiple phosphorylatable residues within these peptides, mass spectra from each identification were submitted to an independent algorithm (Ascore) which assigns confidences to the localization of each phosphorylation.

To investigate if phosphorylation of the evolutionarily conserved filamentous fungal SPRR is also a conserved feature, we isolated *M. grisea* and *N. crassa* CnaA from the two complemented *A. fumigatus* strains and analyzed their *in vivo* phosphorylation status. Two phosphorylations (positions 432 and 436) were detected in the *M. grisea* CnaA SPRR, and a single phosphorylated serine residue (position 423) was detected in the *N. crassa* CnaA SPRR (Figure S3A and S4). Additionally, we also identified the phosphorylation of a serine residue (Ser577) in the C-terminus of *M. grisea* CnaA (Figure S3B). Because we also noted partial hyphal growth complementation with the *M. circinelloides* CnaC construct, which contains a single serine residue (position 404) in the region aligning with the *A. fumigatus* CnaA SPRR, we verified its phosphorylation status *in vivo* and found that this serine residue was phosphorylated, along with another serine residue at position 499 in the C-terminus (Figure S5A and S5B). These results confirmed that CnaA phosphorylation at the SPRR is a unique and conserved mechanism in filamentous fungi.

### The CnaA Serine-Proline Rich Region is phosphorylated by CK1 and Proline directed kinases

In order to determine the potential kinase(s) that may phosphorylate the CnaA SPRR we scanned this region using Scansite 2.0, NetPhos 2.0, and NetPhosK 1.0 programs. These analyses suggested that the amino acids surrounding S406 and S413 of CnaA form a potential consensus sequence for phosphorylation by the proline-directed kinases, such as glycogen synthase kinase (GSK-3), cyclin dependent kinase 1 (CDK1), and mitogen activated protein kinase (MAP Kinase). Casein kinase I (CK1) was also predicted to phosphorylate the SPRR. Based on this prediction, we performed *in vitro* phosphorylation assays using the purified recombinant CnaA regulatory domain (AfRD; regulatory domain spanning 395–482 aa of CnaA, including the SPRR and the CaMBD) from *A. fumigatus* and various combinations of the kinases. The phosphorylation reactions were processed for mass spectrometry after proteolytic digestion to identify the phosphorylated residues. As shown in Table 2, GSK-3β and CK1 alone phosphorylated the S413 and S406 residues, respectively. Because GSK-3β recognizes two substrate motifs characterized by either primed or non-primed phosphorylation sites at serine/threonine-proline rich motifs, and a majority of GSK-3 substrates are formed via prior phosphorylation by an additional kinase at position P+4 (pS/TXXXpS/T) [41], a combination of the two kinases was also tested. Interestingly, GSK-3β and CK1 together phosphorylated all 4 clustered serine residues (S406, S408, S410 and S413) within the SPRR.

**Table 2:** *In vitro* identification of kinases phosphorylating the CnaA SPRR by TiO_2_ enrichment and LC-MS/MS analysis.

Kinase	Peptide Sequence	Phosphorylated Residue	Mascot Ion Score	Ascore Localization probabilty
**GSK-3β**	DETPTSVSPSAP[**pS**]PPLPMDVE	S413	20.4	90%
**CK1**	LEDETPT[**pS**]VSPSAPSPPLPMDVE	S406	15.6	<80%
**GSK-3β+CK1**	DETPT[**pS**]V[**pS**]P[**pS**]APSPPLPMDVE	S406, S408, S410	21.4	<80%, <80%, 99%
	DETPTSVSPSAP[**pS**]PPLPMDVE	S413	32.6	99%
	DETPT[**pS**]VSPSAPSPPLPMDVE	S406	16.5	<80%
	DETPTSVSPSAP[**pS**]PPLPMDVE	S413	18.5	99%
	DETPTSVSP[**pS**]AP[**pS**]PPLPMDVE	S410, S413	25.5	<80%, <80%
	DETPTSVSPSAP[**pS**]PPLPMDVE	S413	33.7	99%
	DETPT[**pS**]VSPSAP[**pS**]PPLPMDVE	S406, S413	21.1	<80%
**GSK-3β and CK1 Inhibitor Treatment**	EELEDETPTSVSPSAP[**pS**]PPLPMDVESSEFK	S413	64.8	99%
	EELEDETPTSV[**pS**]PSAPSPPLPMDVESSEFKR	S408	68.3	80%
	EELEDETPTSVSP[**pS**]AP[**pS**]PPLPMDVESSEFKR	S410, S413	36.9	80%, 99%
**CDK1**	LEDETPTSV[**pS**]PSAPSPPLPMDVE	S408	18.1	80%
	DETPT[**pS**]VSPSAPSPPLPMDVE	S406	20.9	80%
**CDK1+ MAPK**	DETPT[**pS**]VSPSAPSPPLPMDVE	S406	20.4	80%
	DETPTSVSP[**pS**]AP[**pS**]PPLPMDVE	S410, S413	22.7	<80%, 80%
	DETPTSVSPSAP[**pS**]PPLPMDVE	S413	27.9	99%
	LEDETPTSV[**pS**]PSAPSPPLPMDVESSE	S408	36.2	99%
	DETPTSVSPSAP[**pS**]PPLPMDVE	S413	31.3	99%

Recombinant CnaA-AfRD protein was subjected to phosphorylation reactions using the different combination of kinases (GSK-3β, CK1, GSK-3β+CK1, CDK1, MAPK and CDK1+MAPK) and verified for phosphorylation after TiO_2_ enrichment and LC-MS/MS analysis. In addition, the strain expressing CnaA-EGFP was treated with GSK-3β inhibitor (GSK-3β Inhibitor VII) and the CK1 inhibitor (D4476) and the phosphorylation status of CnaA was verified by LC-MS/MS analysis. The uniquely identified phosphorylation residues or combinations of phosphorylation residues are shown. Due to the proximity of multiple phosphorylatable residues within these peptides, mass spectra from each identification were submitted to an independent algorithm (Ascore) which assigns confidences to the localization of each phosphorylation. [pS] indicates the phosphorylated residue.

Next, to determine the role of GSK-3β and CK1 in the phosphorylation of S406, S408, S410 and S413 *in vivo*, we treated the CnaA-EGFP expression strain with GSK-3β and CK1 specific inhibitors, GSK-3β inhibitor VII and D4476, respectively. Both the GSK-3β inhibitor VII and D4476 showed a growth inhibitory effect in a concentration range of 0.5–0.75 µM (data not shown). The CnaA-EGFP fusion protein was isolated after treatment with 0.75 µM each of GSK-3β inhibitor VII and D4476 and analyzed for its phosphorylation status by mass spectrometry. Surprisingly, treatment with GSK-3β and CK1 inhibitors resulted in the dephosphorylation of only S406, but S408, S410 and S413 were phosphorylated, suggesting the possibility of S406 as a target for GSK-3β and CK1 *in vivo*, while other kinases may be involved in phosphorylating the S408, S410 and S413 residues *in vivo*. To investigate this possibility, we performed *in vitro* phosphorylation reactions in presence of the other potential proline-directed kinases, CDK1 and MAP kinase. As shown in Table 2, we found that CDK1 alone phosphorylated 2 serine residues at positions 406 and 408 in the SPRR. Although we could not identify any sites phosphorylated in the presence of MAP kinase alone, a mixture of CDK1 and MAP kinase phosphorylated S406, S408, S410 and S413. Based on these results, it is possible that more than one kinase is responsible for regulating CnaA by phosphorylation at the SPRR *in vivo* and further work on the interaction of these enzymes with CnaA, specifically addressing the timing of phosphorylation of CnaA by these enzymes, will lead to a more definitive understanding of the role of these kinases in the regulation of CnaA.

### FK506 altered the phosphorylation levels of CnaA and CnaB *in vivo*

**Figure 6:**
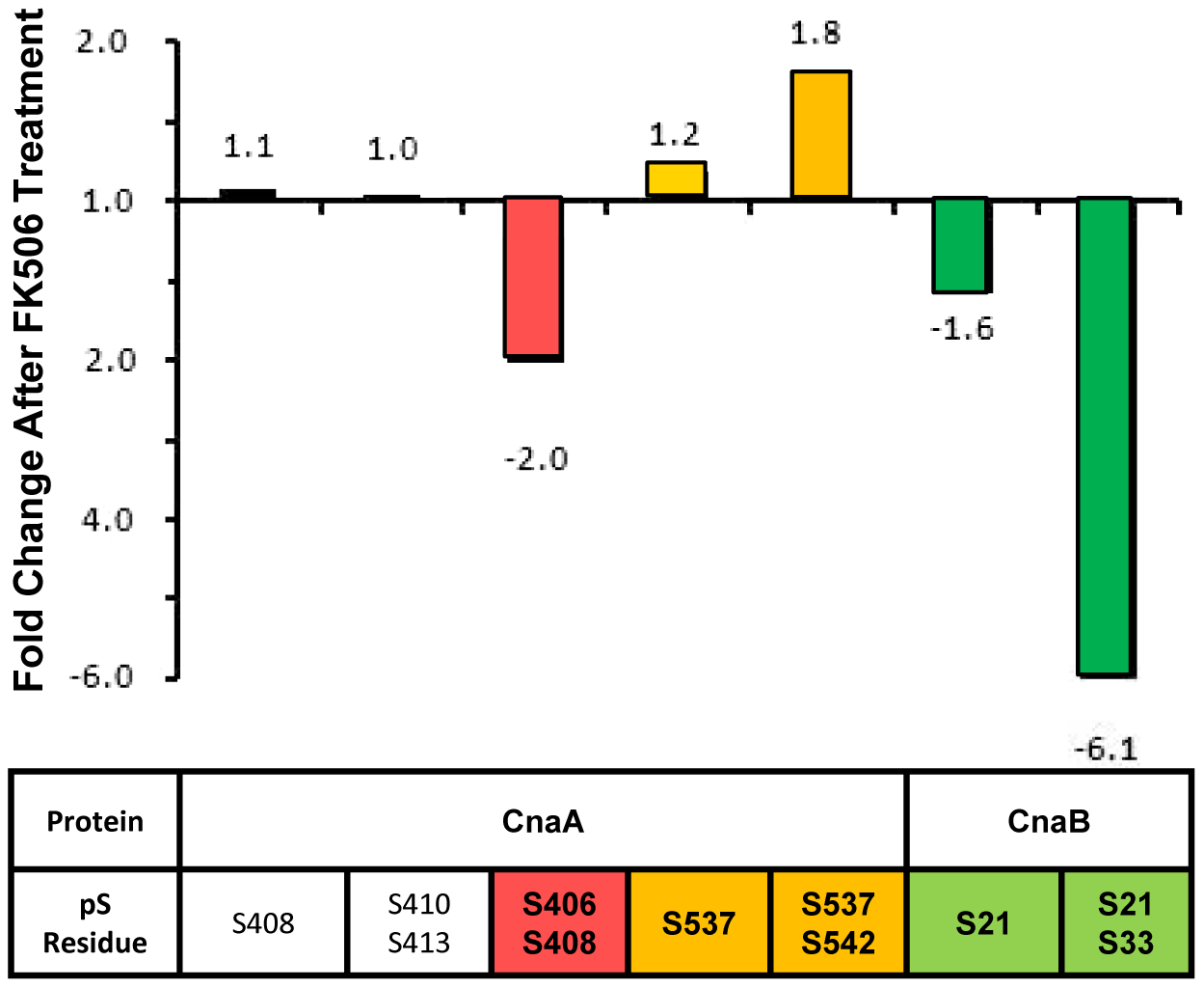
FK506 affects the phosphorylation of Calcineurin complex *in vivo*. Relative fold changes abundance values of individual phosphorylated residues within CnaA and CnaB following TiO_2_ enrichment and LC-MS/MS analysis. Following manual extracted ion chromatogram generation (+/−20 ppm tolerances) of each individual phosphopeptides, peak heights were used to determine abundance, which was subsequently compared across +/− FK506 treated samples. A peak was considered as belonging to the same group across the different samples if it was the same m/z (+/−10 ppm), had the same retention time (+/−30 seconds), and was qualitatively identified as the same species. Raw extracted ion chromatographic peak heights are presented in Supplemental Figures S6 and S7.

Since the immunosuppressant FK506 inhibits calcineurin activity by binding to the immunophilin FKBP12, we also examined the phosphorylation of CnaA and CnaB in the presence of FK506 to correlate phosphorylation versus activity. The FK506-treated sample showed a 2-fold decrease in the phosphorylation of S406 in the CnaA SPRR and a 1.2- and 1.8-fold increase in the phosphorylation of S537 and S542, respectively, in the C-terminus compared to the untreated control (Figure 6 and S6). While CnaB was phosphorylated at S21 and S33 residues under basal conditions, FK506 also significantly reduced the phosphorylation at S33 (Figure 6 and S7). These results suggest a previously unknown link between FK506-FKBP12-mediated inhibition of calcineurin activity and CnaA phosphorylation, including in the novel SPRR. Based on a recent report on the inactivation of GSK-3 by calcineurin inhibitors, cyclosporine A and tacrolimus (FK506) in renal tubular cells [42], and our result demonstrating the phosphorylation of CnaA by GSK-3β and CK1, it is possible that FK506 inhibits the activity of GSK-3β, resulting in its inability to phosphorylate CnaA.

### Phosphorylation of the Serine-Proline Rich Region does not influence changes in conformation following Ca^2+^/calmodulin binding to CnaA

**Figure 7:**
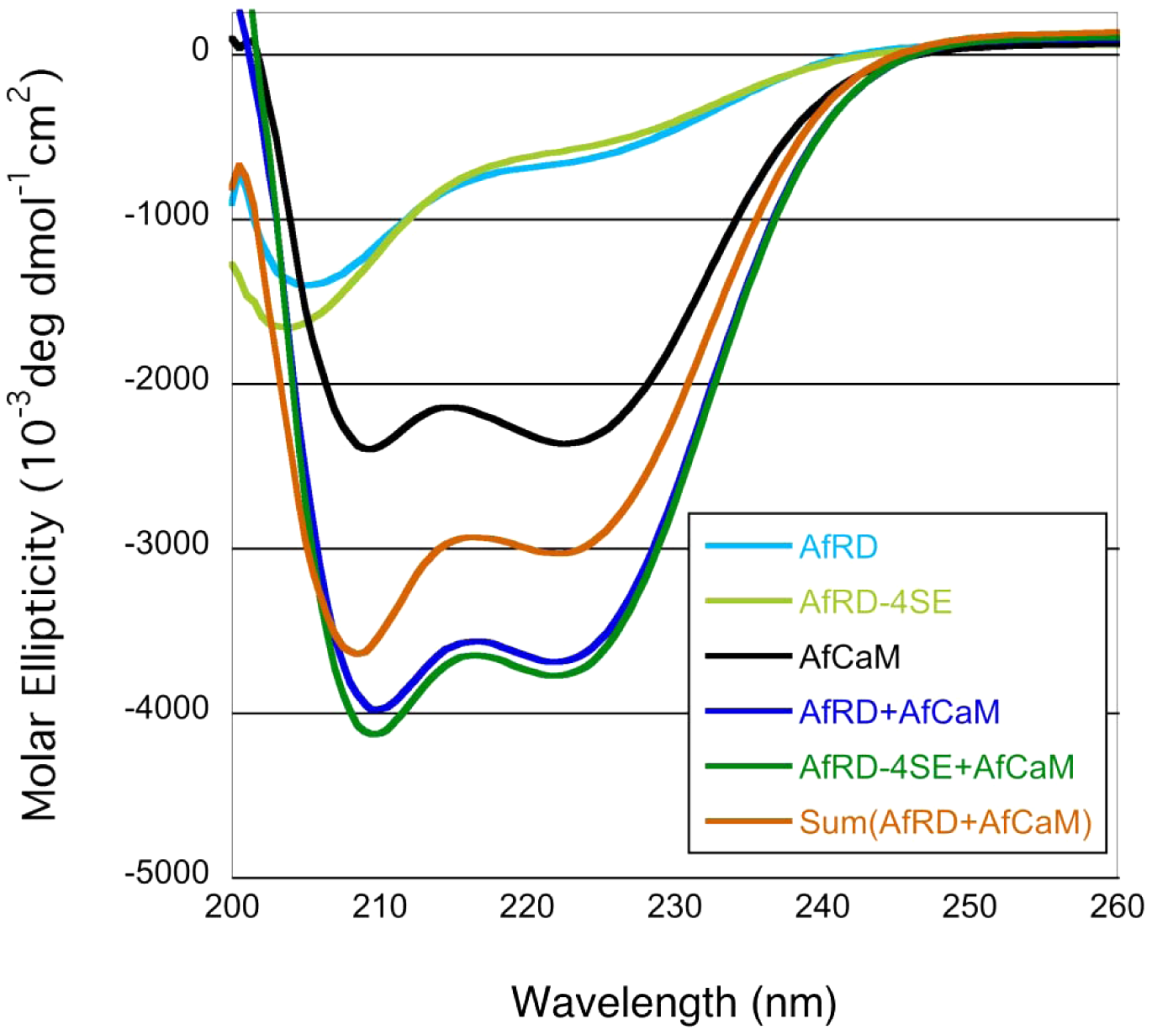
Phosphorylation at the Serine-Proline Rich Region does not influence changes in conformation following Ca^2+^/calmodulin binding. CD spectra collected at 20°C for AfRD and AfRD-4SE, AfCaM, and equimolar mixtures of AfCaM:AfRD and AfCaM:AfRD-4SE. Spectra for AfRD-4SE indicate they occupy similar disordered ensembles. Spectra for the AfCaM:AfRD and AfCaN:AfRD-4SE complexes demonstrate that the phospho-mimetic glutamates in AfRD-4SE do not perturb the secondary structure of the bound RD. Also shown is Sum (AfRD+AfCaM), which is the mathematical sum of the AfRD and AfCaM spectra. This is what would be obtained if the AfRD and AfCaM did not interact.

Ca^2+^/CaM binds to the CaMBD to displace the AID, resulting in calcineurin activation [7]. In mammalian calcineurin, phosphorylation at S411 near the CaMBD resulted in its inactivation [29], while phosphorylation at the same residue in *S. pombe* (S459) activated calcineurin [33]. However, filamentous fungal calcineurins do not show conservation of this phosphorylation site in the CaMBD (Figure S1). A recent study on the structural basis for the activation of human calcineurin by CaM revealed that the intrinsically disordered CaMBD, along with ∼25 to 30-residues adjacent to the AID, adopts an α-helical structure upon Ca^2+^/CaM binding [43]. Since the SPRR is located close to the N-terminal end of the CaMBD (Figure S1), we examined if the phosphorylation of the four serine residues in the SPRR influences Ca^2+^/CaM binding to CnaA or imparts a change in structural content following Ca^2+^/CaM binding. To test this, we utilized the purified AfCaM and the CnaA regulatory domain (AfRD; regulatory domain spanning 395–482 aa of CnaA, including the SPRR and the CaMBD) from *A. fumigatus*. We also expressed another recombinant AfRD in which we mutated the four SPRR serine residues to glutamate to mimic phosphorylated status, designating this as AfRD-4SE. The CD spectra of both complexes (AfRD+AfCaM and AfRD-4SE+AfCaM) indicated essentially identical secondary structural content revealing that conformational changes that occur upon Ca^2+^/CaM binding appear to be unaffected by the phosphorylation (Figure 7). Although glutamate residues may not accurately mimic the phosphorylated state, these results suggest that phosphorylation in the SPRR does not alter the structure of the calcineurin complex, but there is a significant increase in α-helical content. The CD spectrum for AfCaM bound to AfRD has more α-helix than the mathematical sum of the individual AfCaM and AfRD spectra, as evidenced by the stronger negative bands at 222 nm and 208 nm (Figure 7). This is consistent with earlier observations of the human regulatory domain bound to Ca^2+^/CaM [43]. This suggests that the AfRD is disordered, and then folds upon binding to AfCaM, and we speculate that the phosphorylation-dependent activation of CnaA is independent of Ca^2+^/CaM binding.

### Phosphorylation of the Serine-Proline Rich Region is required for proper hyphal growth and virulence but not septal localization

**Figure 8:**
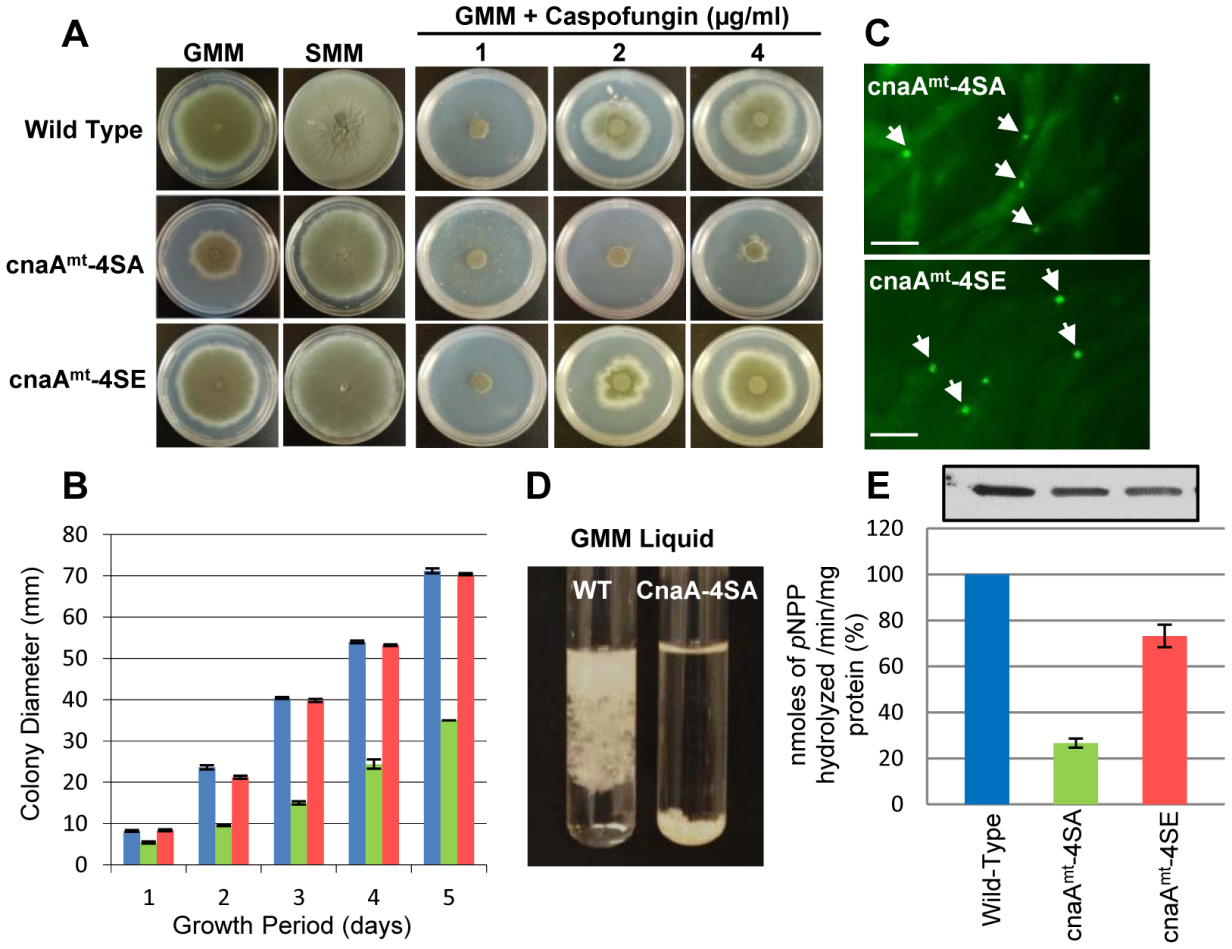
Phosphorylation of CnaA is required for proper hyphal growth. (**A**) Radial growth after 5 days at 37°C on GMM agar and GMM supplemented with 1.2 M sorbitol (SMM) to assess growth remediation. Wild-type, cnaA^mt^-4SA, and cnaA^mt^-4SE strains (1×10^4^ conidia each) were cultured on GMM in varying concentrations of caspofungin for 5 days. The cnaA^mt^-4SA strain, defective in phosphorylation, exhibits a growth defect and lacks caspofungin-mediated paradoxical growth due to lack of calcineurin activity. (**B**) Radial growth is depicted as mean colony diameter in triplicate during 1–5 days growth period. (**C**) Localization of the CnaA mutated constructs by fluorescence microscopy after 24 h growth. (**D**) Growth reduction of the cnaA^mt^-4SA in comparison to the wild-type strain after inoculating 1×10^6^ conidia into GMM liquid and growth for 48 h at 37°C. (**E**) Calcineurin activity after 24 h growth using *p*-nitrophenyl phosphate as a substrate. Data from two separate experiments, each with 6 technical replicates are presented as mean ± SD of nanomoles of *p*NPP released/min/mg protein.

We next mutated the 4 phosphorylated serine residues in the SPRR to alanine (CnaA^mt^-4SA; S406A, S408A, S410A and S413A) to block phosphorylation and also the 4 serine residues to glutamate (CnaA^mt^-4SE) to mimic a fixed phosphorylated state *in vivo*. In comparison to the wild-type strain expressing CnaA-EGFP, only the CnaA^mt^-4SA strain exhibited a growth defect (Figure 8A, 8B, and 8D; GMM panel), but CnaA septal localization remained unaltered (Figure 8C). To verify if the slightly increased cytosolic staining seen in the CnaA^mt^-4SA strain is due to protein instability, we performed Western analysis of the extracts obtained from the strains. As shown in Fig. 8E (upper panel), all the mutated constructs were stably expressed, indicating the possibility that the higher cytosolic distribution seen in the CnaA^mt^-4SA could be a consequence of this mutation, leading to some amount of CnaA mislocalization. While the control strain and the CnaA^mt^-4SE strain showed complete hyphal growth, the CnaA^mt^-4SA strain had very poor growth in liquid media (Figure 8D). This indicated that the phosphorylation of the 4 serine residues is required for calcineurin-mediated regulation of hyphal growth but is not required for CnaA septal localization. Moreover, the CnaA^mt^-4SA strain showed hyphal growth recovery in the presence of sorbitol, indicative of osmotic stress and cell wall defects (Figure 8A; SMM panel) similar to our other calcineurin mutant strains [3], [36], [37], [44]. The CnaA^mt^-4SA strain was also hypersensitive to caspofungin (Figure 8A; GMM+Caspofungin panel). Supporting these observations, calcineurin activity was also decreased by ∼70% in the CnaA^mt^-4SA strain compared to the wild-type strain, indicating that phosphorylation plays an important role in the regulation of calcineurin activity (Figure 8E; lower panel). Although the CnaA^mt^-4SE strain showed complete recovery of hyphal growth, its activity was also decreased by ∼25%, indicating the possibility of active phosphorylation-dephosphorylation events required to fully control calcineurin activation and function. Although the substitution of glutamate residues for phosphorylated serines does not perfectly mimic phosphorylation *in vivo*, the CnaA^mt^-4SE strain indeed showed wild-type comparable hyphal growth, but only a slight decrease in calcineurin activity. It is possible that a threshold level of calcineurin activity is sufficient for regular hyphal growth.

**Figure 9:**
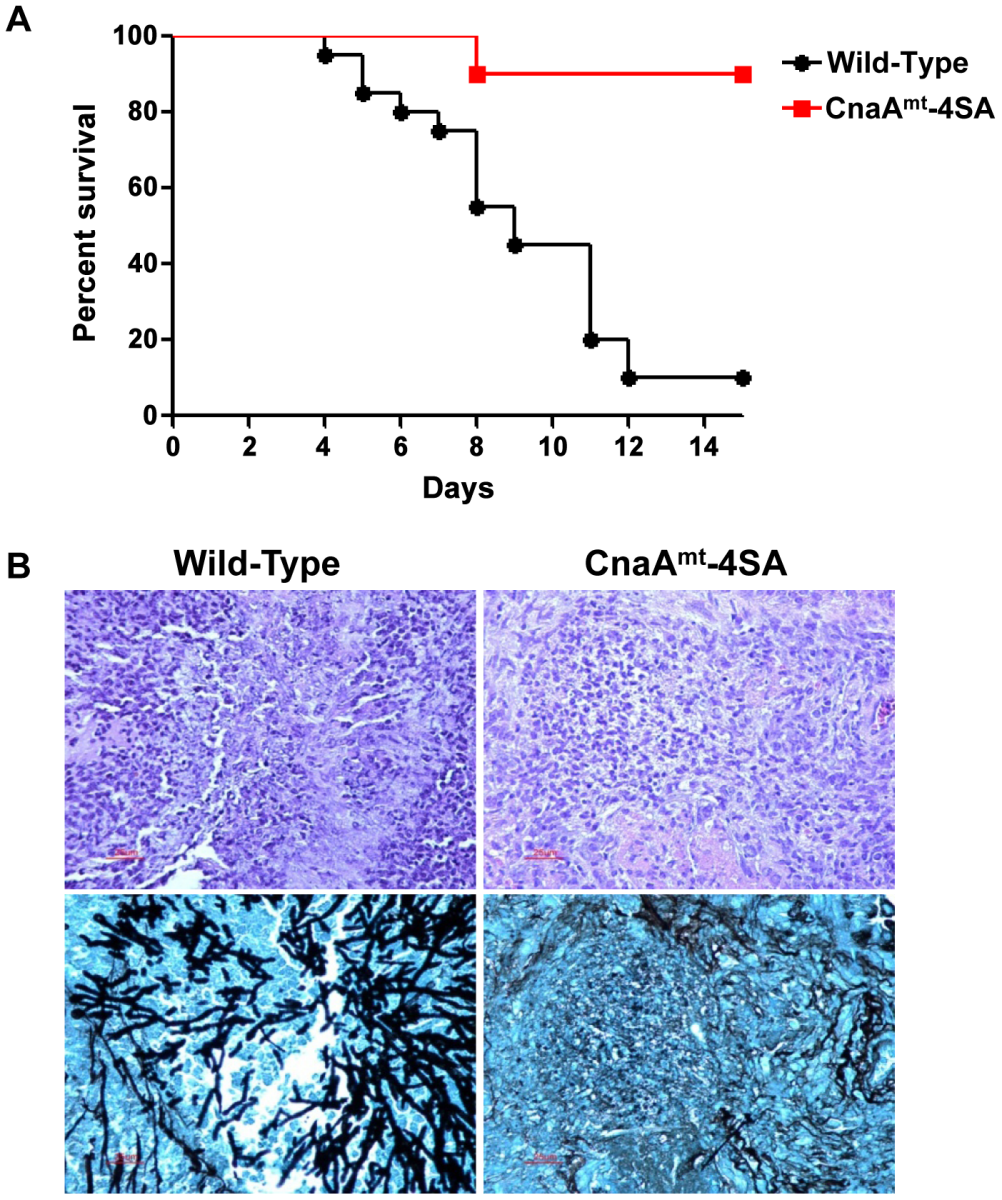
Mutation of the phosphorylated residues in the SPRR cause reduced virulence. (A) Kaplan-Meier survival curve showing virulence analysis in a murine inhalational model of invasive aspergillosis. At day 14 post-infection, mice infected with the WT strain displayed 90% mortality, in contrast to the cnaA^mt^-4SA strain which exhibited a significant reduction in virulence (*P*<0.0001). (B) Histopathological examination of the lungs shows less inflammation in the cnaA^mt^-4SA strain as well as significantly less hyphal invasion.

Because we noted a significant growth defect of the CnaA^mt^-4SA strain (Figure 8), we examined its virulence in our persistently neutropenic murine inhalational model of invasive aspergillosis. The mortality associated with CnaA^mt^-4SA strain infection (Figure 9A) was significantly lower (10%) in comparison to the wild-type strain (90%) (P<0.0001) indicating that phosphorylation of the four serine residues clustered in this novel SPRR is critical for calcineurin function and virulence. Consistent with the survival data, lung histopathologic studies revealed both decreased inflammation as well as a near absence of hyphal growth in the mice infected with the CnaA^mt^-4SA strain compared to those infected with the wild-type strain (Figure 9B).

### CnaA septal localization is independent of Ca^2+^/calmodulin binding but requires the PxIxIT binding motif

**Figure 10:**
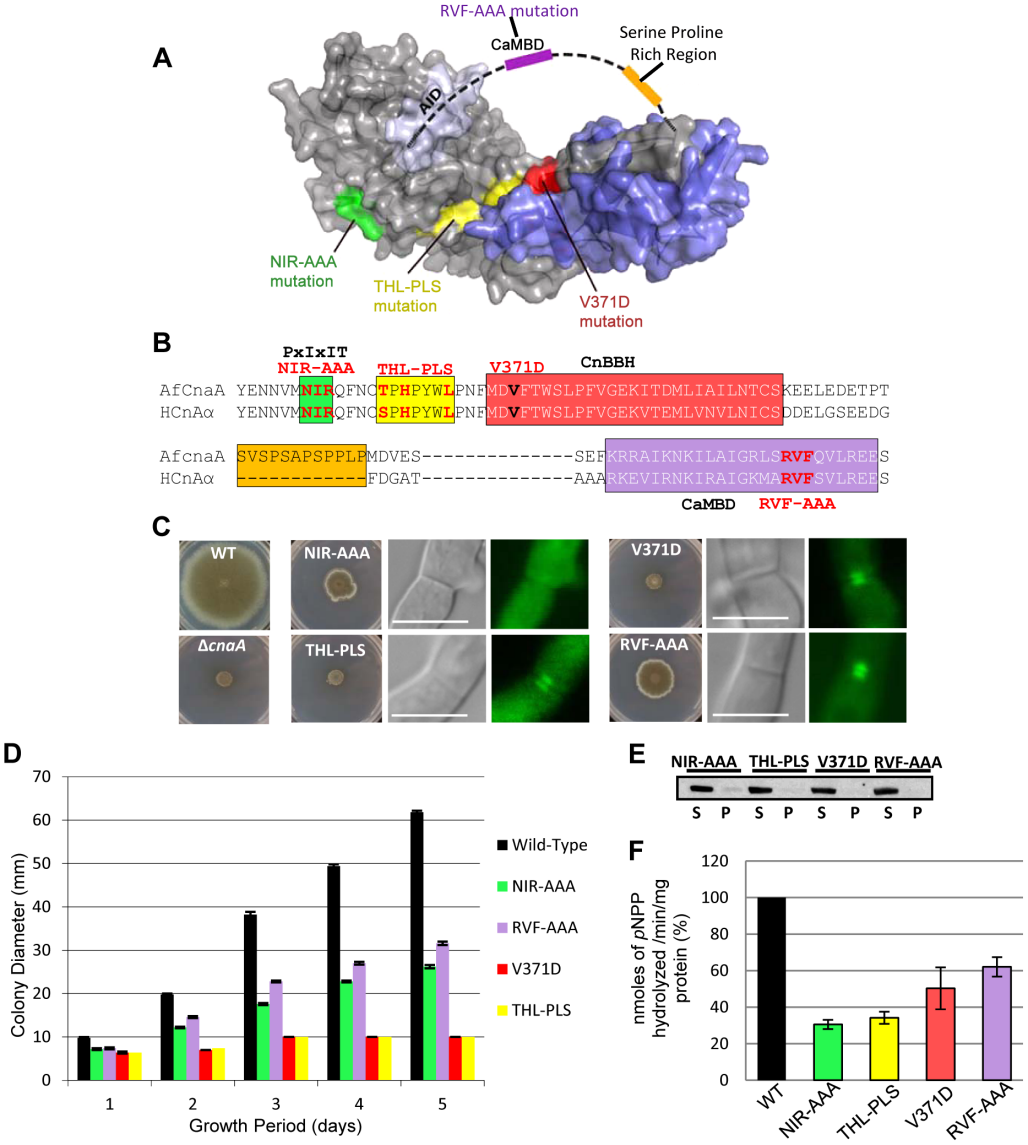
Septal localization of CnaA is independent of Ca^2+^/calmodulin binding but requires the PxIxIT binding motif. (**A**) Homology models for the *A. fumigatus* CnaA (gray) and CnaB (blue) subunits reveal relative arrangement of subdomains and mutated regions. The Serine Proline Rich Region (SPRR, orange) and calmodulin binding domain (CaMBD, purple) are indicated schematically, as they are likely disordered. (**B**) Alignment of *A. fumigatus* CnaA and human CnA-α subunit including the PxIxIT, CnBBH and the CaMBD. (**C**) Radial growth after 5 days at 37°C on GMM agar. CnaA septal localization after 24 h growth (**D**) Radial growth is depicted as mean colony diameter in triplicate over 5 days growth. (**E**) Western detection of CnaA-EGFP fusion proteins using anti-GFP polyclonal antibody and peroxidase labeled anti-rabbit IgG secondary antibody. S and P indicate supernatant and pellet fractions, respectively. (**F**) Calcineurin activity was determined using *p*-nitrophenyl phosphate as a substrate. Data from two separate experiments, each with 6 technical replicates are presented as mean ± SD of nanomoles of *p*NPP released/min/mg protein.

Binding studies with the human calcineurin-NFAT complex previously revealed the PxIxIT motif as a common binding site for calcineurin on its substrates [38]. In *S. cerevisiae*, mutation of the calcineurin residues (N366 I367 R368) in contact with the PxIxIT motif resulted in defective substrate interaction [45]. Recent structural studies of Ca^2+^/CaM bound to a 25-residue peptide spanning the CaMBD in the human calcineurin catalytic subunit also revealed that R408, V409, and F410 play a major role in rigidity and stabilization of the central helix of CaM bound to calcineurin [46]. To investigate the role of these specific domains for CnaA septal localization and calcineurin function in *A. fumigatus*, we mutated the PxIxIT-binding NIR residues to alanines (NIR-AAA), as well as the critical Ca^2+^/CaM-binding RVF residues in the CaMBD to alanines (RVF-AAA; Figure 10A and 10B). The NIR-AAA mutation only partially restored hyphal growth and completely mislocalized CnaA, indicating that septal localization of CnaA occurs through binding to other protein(s) (Figure 10C and 10D). On the contrary, the RVF-AAA mutation had partial hyphal growth restoration but did not affect CnaA septal localization (Figure 10C and 10D), supporting our CnaA truncation results. Western analysis confirmed that both mutations maintained protein stability (Figure 10E). The observed growth defect with the RVF-AAA mutation may be due to the inability of CaM to bind to CnaA, and as a result the AID remains bound to the regulatory domain, leading to continued inhibition of calcineurin activity. Although CaM localizes at the hyphal tip and septum in *A. nidulans* [47], and we confirmed this in *A. fumigatus* (data not shown), these results, coupled with our truncational analyses, confirmed that CnaA localization at the septum is CaM-independent, yet CaM is required to activate CnaA to completely restore hyphal growth.

### Mutations in the CnaA catalytic active site and the CnaB-binding helix do not alter septal localization of CnaA

Critical regions controlling calcineurin function in *S. cerevisiae* have been identified by substitution of V385 with an aspartic acid that disrupted the interaction between the catalytic and the regulatory subunit, and also by random mutagenesis of three residues (S373, H375, and L379) that led to loss of calcineurin activity but did not disrupt calcineurin A binding to Ca^2+^/CaM or to calcineurin B [48]. To examine if any of these mutations would affect the septal localization or function of *A. fumigatus* CnaA, we mutated V371 to aspartic acid (V371D) and the T359, H361, and L365 to proline, leucine and serine (THL-PLS), respectively. Both mutations had a significant effect on hyphal growth, but neither affected CnaA septal localization (Figure 10C and 10D). The V371D mutation confirmed our previous finding [36] that, although CnaB is not required for CnaA septal localization, it is required for CnaA function and growth. The THL-PLS mutation had an effect on the catalytic activity and therefore it is possible that although CnaA is localized at the hyphal septum it is catalytically inactive. We confirmed the stability of each mutation by Western analysis (Figure 10E). The reduction in calcineurin activity due to these mutations (Figure 10F) and the lack of paradoxical growth recovery (Figure S8) established that catalytic site residues and CnaB-binding activity of CnaA do not influence its septal localization, yet catalytically active calcineurin is required at the hyphal septum to direct proper hyphal growth.

## Discussion

Calcineurin inhibitors are promising new antifungal candidates due to their unique mode of action from other antifungal classes (e.g., polyenes, triazoles, echinocandins), efficacy against emerging resistant strains, and synergism with existing antifungals [4]. However, currently-used calcineurin inhibitors complex with immunophilins leading to host immunosuppression [49]. Although calcineurin has been well studied in several organisms and its functional domains described, few studies have focused on mutations in its key domains *in vivo*, and none have examined phosphorylation as a mechanism of calcineurin function in a human pathogen. By deleting the C-terminal regulatory domains of CnaA which led to progressive defects in hyphal growth (Figure 1 and 2), we identified a unique fungal-specific 23 residue linker domain between the CnBBH and the CaMBD, containing the novel and evolutionarily conserved SPRR (Figure 3A). Inclusion of the SPRR showed full recovery of hyphal growth, concomitant increase in calcineurin activity, and clear localization of CnaA to the hyphal septum (Figure 4).

To evaluate the conservation of this linker region, we examined it in 22 eukaryotes selected based on divergence and to include model organisms and pathogens affecting both humans and plants (Figure S1). Phylogenic analysis showed that the linker containing the SPRR clearly distinguished the filamentous fungi (Figure 3B). Based on the CnaA-CnaB molecular model we created (Figure 10A), the SPRR is present outside the core binding region between CnaA and CnaB, and the preponderance of proline and serine residues in this linker creates a hydrophobic environment which could lead to binding to other as of yet unknown proteins.

To determine the importance of the SPRR for CnaA function *in vivo*, we performed complementation tests in our *A. fumigatus* Δ*cnaA* mutant strain with *cnaA* homologs from human, *S.cerevisiae*, *C. neoformans* and *M. circinelloides*, all of which lacked similarity within the SPRR (Figure 4A and 4B). While neither human nor *S.cerevisiae* CNA, which possess an overall 56% and 50% similarity to *A. fumigatus* CnaA, respectively, complemented the hyphal growth defect (data not show), *C. neoformans* CNA1, which exhibits 67% similarity to *A. fumigatus* CnaA, localized to the septum but did not restore hyphal growth (Figure 4B). Interestingly, only *M. circinelloides* CnaC, which had partial similarity to the *A. fumigatus* SPRR, and exhibits 59% similarity to *A. fumigatus* CnaA, partially complemented the hyphal growth defect and localized CnaA at the septum (Figure 4A and 4B). Domain swapping of the *C. neoformans* CNA1 C-terminus with the *A. fumigatus cnaA*, to include the SPRR in the chimera, completely restored hyphal growth and localized CnaA at the septum (Figure 4B), revealing that the SPRR is required for calcineurin function in regulating proper hyphal growth. To further confirm this, calcineurins from more closely related ascomycete filamentous fungi, such as *N.crassa* and *M. grisea*, each of which exhibit overall similarity of 80% and greater conservation in the SPRR, were used for complementation of the *A. fumigatus* Δ*cnaA* strain. The respective complemented strains showed proper septal localization and complete hyphal growth recovery (Figure 4C). Taken together, although these results clearly indicated the importance of the SPRR for calcineurin function, we cannot exclude the possibility that some minor variations in other regions of calcineurin may also limit the ability of calcineurins from other species to fully complement the *A. fumigatus* Δ*cnaA* strain.

Our phosphoproteomic analyses provided unequivocal evidence of *A. fumigatus* CnaA phosphorylation *in vivo* at the unique SPRR that is specific to filamentous ascomycetes (Figure 5 and Table 1). Phosphorylation at the SPRR serine residues in proximity to the proline residues may induce secondary structure conformation in the molecule facilitating binding to other proteins. Moreover, as this SPRR is non-conserved in the yeasts and is completely absent in the human calcineurin α-subunit, it may have been acquired during evolution by diverging from the phylum basidiomycota.

As mentioned earlier to confirm if phosphorylation at the SPRR is also important for other filamentous fungi, we performed similar complementation analyses with the closely related filamentous fungi *N. crassa* and *M. grisea*, which contain 4 and 5 serine residues in their SPRR, respectively, and both complemented hyphal growth (Figure 4C). Phosphoproteomic analyses confirmed the phosphorylation of serine residues from those fungi within the SPRR (Figure S3 and S4), demonstrating a unique feature of calcineurin function via a conserved phosphorylation in this novel domain found only in filamentous fungi. Mutation of the 4 serine residues in the *A. fumigatus* SPRR to block phosphorylation of CnaA caused increased branching and reduction in hyphal growth, confirming the significance of this phosphorylation (Figure 8). Most importantly, we also found that the CnaA^mt^-4SA strain was defective in virulence (Figure 9), strengthening our results of the requirement for phosphorylation at the SPRR for calcineurin activation and function *in vivo*.

Phosphorylation was reduced in both the catalytic and the regulatory calcineurin subunits following treatment with FK506 (Figure 6, S6 and S7). It is possible that the FK506-FKBP12 complex may indirectly cause an inhibitory effect on the kinase that phosphorylates calcineurin at the SPRR and also at the N-terminus of CnaB. Based on the phosphorylation of residues in both the SPRR and the C-terminus, we speculated that more than one kinase is responsible. Although mammalian calcineurin was shown to be phosphorylated at S411 in the CaMBD by PKC and CaM kinase II 29–31, and the same residue at position S459 in *S. pombe* was phosphorylated by Cds1 check point kinase [33], this serine residue is not conserved among filamentous fungal calcineurins. Based on our identified phosphorylation sites in *A. fumigatus* CnaA and the SPRR, we were able to predict the phosphorylation of this region by potential proline-directed kinases such as GSK-3, CDK1 and MAP kinase. By *in vitro* phosphorylation assays, using the enzymes GSK-3β and CK1, we identified that all 4 serine residue in the SPRR were phosphorylated. Additionally, S413 and S408 are both flanked by downstream proline residues, which represent typical GSK-3 phosphorylation sites. While GSK-3β alone phosphorylated S413 in the SPRR, CK1 alone phosphorylated S406, and a combination with GSK-3β led to phosphorylation of other serine residues (S408 and S410), revealing that the prephosphorylation of S406 residue by CK1 may trigger the subsequent phosphorylation of S408 and S410. Interestingly, a previous study showed that the yeast Mck1 protein kinase belonging to the GSK-3 kinase family stimulated calcineurin activity by phosphorylating Rcn1, and in the absence of GSK-3 kinase, calcineurin activity was fully inhibited revealing a allosteric mechanism of calcineurin regulation by Rcn1 [50]. We further validated our *in vitro* phosphorylation data by examining the phosphorylation status of CnaA *in vivo* after treating with inhibitors for GSK-3β and CK1, which confirmed the absence of phosphorylation at S406 only but not the other serine residues, leading to the notion that other kinases may also phosphorylate the CnaA SPRR *in vivo*. The observed phosphorylation of CnaA SPRR by CDK1 and MAP kinase *in vitro* strengthens this possibility. However, further analyses are required to specifically understand how these enzymes actually regulate CnaA.

Since the CaMBD is in close proximity to the SPRR, we hypothesized that phosphorylation in the SPRR caused conformational changes or altered binding between Ca^2+^/CaM and the calcineurin complex. Circular dichroism studies revealed that conformational changes that occur after Ca^2+^/CaM binding remained unaffected between unphosphorylated and phosphomimetic SPRR constructs (Figure 7), indicating that CaM-mediated activation of calcineurin is independent of SPRR phosphorylation. This is intriguing when considering that calcineurin phosphorylation at S411 in Rat slightly decreased the affinity of calcineurin for Ca^2+^/CaM, causing its inactivation [29], and phosphorylation at the same residue (S459) in *S. pombe* activated calcineurin [33].

**Figure 11:**
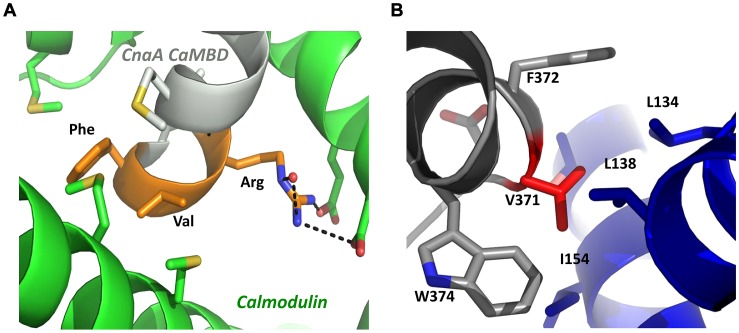
Model of the CaMBD and the CnaB-binding pocket in *A.* **fumigatus**** **CnaA.** **** (**A**) The RVF sequence (orange) in the CnaA CaMBD (gray) interacts specifically with calmodulin (green). The arginine residue makes several important salt bridges to aspartic acid residues in calmodulin, which would be lost upon mutation to alanine. The valine and phenylalanine residues bind in a hydrophobic pocket comprising several methionine residues. Mutation of these residues to alanine would result in the loss of important van der Waals interactions and significantly destabilize the calcineurin-calmodulin interaction. (**B**) V371 lies in a hydrophobic pocket at the interface between CnaA and CnaB subunits. V371D would unfavorably place a charged residue in this pocket.

Surprisingly, even though Ca^2+^/CaM is well known to bind to calcineurin and localize at the hyphal tips and septum [47], the septal localization of CnaA was not impaired by the deletion of the C-terminus (Figure 1), indicating that CaM is not involved in CnaA septal localization. We confirmed this by mutating key residues (RVF-AAA) within the CaMBD (Figures 10 and 11A), which did not cause CnaA mislocalization from the septum but affected hyphal growth, indicating the requirement of CaM for calcineurin activity and growth but not for septal localization.

**Figure 12:**
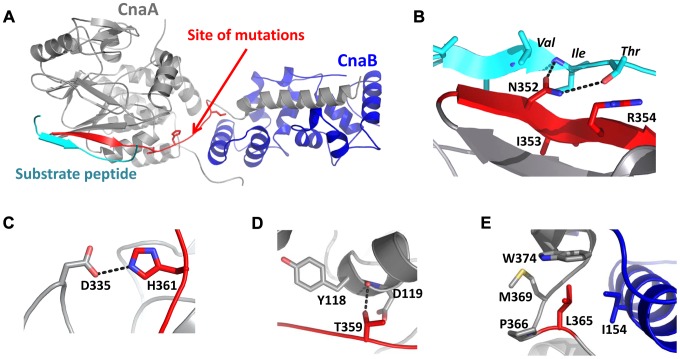
Molecular modeling of *A.* **fumigatus**** **CnaA substrate recognition motif.** **** (**A**) Model of the *A. fumigatus* CnaA (gray) and CnaB (blue) subunits, indicating relative positions of substrate peptide (cyan) and sites on the protein where mutations are found (red). The Phyre server was used to prepare homology models using the X-ray structure of the human calcineurin heterodimer bound to a substrate peptide (PDB ID 2P6B). Most mutations are predicted to destabilize portions of the protein required for heterodimer formation or substrate recognition. (**B**) Interactions of CnaA subunit with substrate peptide of sequence PVIVIT. N352A mutation would disrupt several hydrogen bonds that stabilize this interaction. (**C**) H361 forms interactions (including a likely hydrogen bond with D335) that are important for maintaining the structure of CnaA near the substrate binding region. H361L mutation would abolish these interactions and place a hydrophobic residue in a fairly solvent-exposed portion of the protein. (**D**) As with H361, T359 is important for structural stability near the substrate binding region of CnaA. T359P mutation would disrupt these important interactions. (**E**) L365 lies in a hydrophobic pocket at the interface between CnaA and CnaB subunits; L365S mutation would be unfavorable for heterodimer formation.

Mutation of V371 (V371D), located at the beginning of the long helix that forms the bulk of the CnaB binding interface of CnaA, would place a charged residue in an unfavorable hydrophobic environment and result in destabilization of the calcineurin heterodimer (Figure 11B). Mutation of N352, I353, and R354 (NIR-AAA) residues that lie in the substrate recognition β strand of CnaA (Figures 12A and 12B) [38], [45], revealed that CnaA localizes at the septum (Figure 10) by likely binding to other proteins, and its septal localization is important for hyphal growth. Neither the V371D mutation, nor the triple mutation of the residues T359, H361, and L365 (THL-PLS) that lie in a stretch of amino acids connecting the last β strand of the catalytic core domain of CnaA with the helical CnaB binding domain (Figures 10, and 12C, 12D and 12E), altered the CnaA septal localization. This reconfirmed that CnaA localizes to the septum independent of CnaB, validating our previous findings [36]. The septal localization aspect of calcineurin complex is specific to filamentous fungi. Although the exact mechanism of how these mutations affect calcineurin function at the hyphal septum is unknown, we expect that the availability of the crystal structure for *A. fumigatus* calcineurin in the future would help us in better understanding the consequence of these mutations. Based on our previous report [36], we presume that the localization and activity of the calcineurin complex at the septum is necessary to maintain proper septum formation through proper cell wall assembly at the septum by regulating the enzymes involved in β-glucan and chitin synthesis, apart from also regulating the cell wall repair mechanisms at the hyphal septa under stress. In contrast to the yeast cells, filamentous fungi proliferate by hyphal tip extension and form septa that divide the hyphal compartments at regular intervals; during these two very important processes, active cell wall biosynthesis is required. Based on our previous findings and also our present results, we think the calcineurin phosphorylation is important for not only its activation but also for its interaction with other substrates that are necessary for cell wall biosynthesis. Our findings on *A. fumigatus* CnaA are broadly significant because of the conservation of this unique SPRR among filamentous fungi, and phosphorylation in this region is a newly described mode of calcineurin regulation for growth and virulence. Future studies directed towards understanding the phosphorylation status of CnaA at different stages of growth, stress conditions, and also identifying phosphorylation-dependent interactions of the calcineurin complex with other substrates *in vivo* will help reveal the exact mechanism of calcineurin-mediated regulation of hyphal growth in filamentous fungi. In addition, given the importance of CnaA phosphorylation at the SPRR for its activity, function, and virulence in this pathogen, and the absence of the SPRR in human calcineurin, future antifungal drug targeting to combat invasive aspergillosis could exploit the SPRR.

## Materials and Methods

### Ethics statement

Animal studies at Duke University Medical Center were in full compliance with all of the guidelines of the Duke University Medical Center Institutional Animal Care and Use Committee (IACUS) and in full compliance with the United States Animal Welfare Act (Public Law 98-198). Duke University Medical Center IACUC approved all of the vertebrate studies under the protocol number A-038-11-02. The studies were conducted in the Division of Laboratory Animal Resources (DLAR) facilities that are accredited by the Association for Assessment and Accreditation of Laboratory Animal Care (AAALAC).

### Strains, culture conditions and calcineurin mutations

Strains are listed in Table S1. Δ*cnaA* mutant strain (Δ*cnaA*::*A. parasiticus pyrG*) [3], was used for transformations. Isogenic *A. fumigatus* wild-type strain (AF293) or the strain expressing *cnaA*-*egfp* under its native promoter [36] were used as control strains for radial growth assays and paradoxical growth testing [36], [37]. Axioskop 2 plus microscope equipped with AxioVision 4.6 imaging software was used for fluorescence microscopy [36]. For Δ*cnaA* mutant complementation analyses, the respective truncated or mutated cDNAs encoding *cnaA* amplified by using pUCGH-*cnaA* as a template with primers listed in Table S2 were cloned in the plasmid pUCGH-*cnaApromo* and transformants selected in the presence of hygromycin B [36] were verified by Southern analysis.

### Cloning of calcineurin A genes from other organisms

The calcineurin A encoding cDNAs from *C. neoformans*, *M. grisea*, *N. crassa* and *M. circinelloides* were amplified from respective cDNA libraries. All genes were amplified using the respective templates and primers listed in Table S2 and cloned into the pUCGH vector as previously described [36]. For cloning human *CNA-α* subunit the plasmid, pET15b CnA CnB (obtained from Addgene), was used as a template. *S. cerevisiae* genomic DNA was used as a template to amplify *S. cerevisiae CNA1*. The plasmids were sequenced to verify for accuracy prior to their transformation into the *A. fumigatus* Δ*cnaA* mutant strain. Transformants were selected in the presence of hygromycin B (150 µg/ml).

### Protein extraction for Western analysis and calcineurin activity

Preparation of cell extracts and Western detection were performed as described earlier [36]. Cell extracts from 24 h cultures were assayed for calcineurin phosphatase activity [36] using *p*-nitrophenyl phosphate as substrate at 405 nm. The difference of absorbance values between the amounts of *p*-nitrophenol released in the strains versus the Δ*cnaA* Δ*cnaB* double mutant control strain represented the phosphatase activity mediated by calcineurin. Each experiment consisted of two biologic replicates, with each assay consisting of 6 technical replicates; data are presented as mean ± SD of nanomoles of *p*NPP released/min/mg protein.

### Calcineurin A purification and nano-flow Liquid Chromatography Electrospray Ionization Tandem Mass Spectrometry (LC-MS/MS) analysis

Total cell lysates were extracted and normalized to contain ∼10 mg protein in each sample before GFP-Trap® affinity purification (Chromotek) and processed for TiO_2_ phosphopeptide enrichment and mass spectrometry as previously described [51]. The dried phospho-peptide enriched samples were resuspended in 10 µl of 2% acetonitrile, 0.1% formic acid, 10 mM citric acid and subjected to chromatographic separation on a Waters NanoAquity UPLC equipped with a 1.7 µm BEH130 C_18_ 75 µm I.D.×250 mm reversed-phase column. The mobile phase consisted of (A) 0.1% formic acid in water and (B) 0.1% formic acid in acetonitrile. Following a 5 µl injection, peptides were trapped for 5 min on a 5 µm Symmetry C_18_ 180 µm I.D.×20 mm column at 20 µl/min in 99.9% A. The analytical column was held at 5% B for 5 min then switched in-line and a linear elution gradient of 5% B to 40% B was performed over 90 min at 300 nl/min. The analytical column was connected to a fused silica PicoTip emitter (New Objective, Cambridge, MA) with a 10 µm tip orifice and coupled to an LTQ-Orbitrap XL mass spectrometer. In some experiments the analytical column was connected to a fused silica PicoTip emitter (New Objective, Cambridge, MA) with a 10 µm tip orifice and was coupled to a Waters Synapt G2 QToF mass spectrometer through an electrospray interface operating in a data-dependent mode of acquisition. The instrument was set to acquire a precursor MS scan in the Orbitrap from *m/z* 400–2000 with r = 60,000 at *m/z* 400 and a target AGC setting of 1e6 ions. In a data-dependent mode of acquisition, MS/MS spectra of the three most abundant precursor ions were acquired in the Orbitrap with r = 7500 at m/z with a target AGC setting of 2e5 ions. Max fill times were set to 1000 ms for full MS scans and 500 ms for MS/MS scans with minimum MS/MS triggering thresholds of 5000 counts. For all experiments, fragmentation occurred in the LTQ linear ion trap with a CID energy setting of 35% and a dynamic exclusion of 60 s was employed for previously fragmented precursor ions. When using the Waters Synapt G2 QToF mass spectrometer through an electrospray interface operating in a data-dependent mode of acquisition the instrument was set to acquire a precursor MS scan from *m/z* 50–2000 with MS/MS spectra acquired for the three most abundant precursor ions. For all experiments, charge dependent CID energy settings were employed and a 120 s dynamic exclusion was employed for previously fragmented precursor ions.

### Qualitative identifications and selected ion chromatograms from raw LC-MS/MS data

Raw LC-MS/MS data files were processed in Mascot distiller (Matrix Science) and then submitted to independent Mascot database searches (Matrix Science) against a SwissProt (fungus taxonomy) containing both forward and reverse entries of each protein. Search tolerances for LTQ-Orbitrap XL data were 10 ppm for precursor ions and 0.02 Da for product ions, and for Synapt G2 data were 10 ppm for precursor and 0.04 Da for product ions using trypsin specificity with up to two missed cleavages. Carbamidomethylation (+57.0214 Da on C) was set as a fixed modification, whereas oxidation (+15.9949 Da on M) and phosphorylation (+79.9663 Da on S, T, and Y) were considered a variable modification. All searched spectra were imported into Scaffold (Proteome Software) and protein confidence thresholds were set using a Bayesian statistical algorithm based on the PeptideProphet and ProteinProphet algorithms which yielded a peptide and protein false discovery rate of 1%. Phosphopeptide intensities were obtained by generating selected ion chromatograms (20 ppm window around most abundant charge state of precursor ion with seven point Boxcar smoothing) from raw LC-MS data. MS response at peak apex was used for quantitating abundance.

### *A. fumigatus* calcineurin A-AfRD, calmodulin and circular dichroism

*E. coli* optimized genes for *A. fumigatus* calcineurin (AfRD; regulatory domain from 395–482 aa of CnaA including the SPRR and the CaMBD) and calmodulin (AfCaM) were synthesized and ligated into a pUC57. The genes were digested and ligated into the pET303/CT-His vector using XhoI and XbaI. The mutant AfRD4Ser-Glu (AfRD-4SE), containing mutations S14E, S16E, S18E, and S21E, was made using a QuikChange Site-Directed Mutagenesis Kit. AfRD and AfRD-4SE were transformed into *E. coli* BL-21 (DE3) cells for expression, and purified on a Ni^2+^/NTA column followed by a calmodulin-sepharose column. AfCaM was purified on a 2-trifluoromethyl-10-aminopropylphenothiazine (TAPP)-sepharose column. Protein concentrations were determined by bicinchoninic acid assay. Circular dichroism (CD) experiments were performed using a Jasco J-810 spectropolarimeter. Sample buffers were composed of 20 mM Tris, 200 mM NaCl, 4 mM EGTA, pH 7.5, and 20 mM CaCl_2_. Samples contained AfRD, AfCaM, or equimolar concentrations of AfRD and AfCaM. The CD experiments shown were all performed in the presence of an excess of calcium in order to determine the effects of the phospho-mimics on the AfRD conformation when bound by AfCaM. The concentrations of either protein alone per sample ranged from 10–20 µM. In the sample that contained both of AfRD and AfCaM, the total protein concentration per sample ranged from 10–25 µM. Spectra were collected in quartz 1 mm pathlength cuvettes. Samples were scanned from 200–260 nm in 0.5 nm increments at a scanning speed of 50 nm/sec and each spectrum is the average of 4 scans. The raw CD data (in millidegrees) was converted to molar ellipticity. The amount of secondary structure was determined using the CONTIN/LL deconvolution program.

### *In vitro* phosphorylation assays

*In vitro* phosphorylation reactions with GSK-3β, CK1, CDK1/cyclinB and MAP kinase (New England Biolabs) contained 10 µg of recombinant CnaA-AfRD protein, reaction buffer supplied by the manufacturer, and 500 µM ATP in total volume of 50 µl. GSK-3β (2500 U), CK1 (5000 U), CDK1 (100 U) and MAP kinase (500 U) were used for each reaction. The reactions were performed either with single enzymes or combinations of the different enzymes. The reactions were incubated at 30°C for 4 h and processed for mass spectral analysis following digestion with Glu-C and TiO_2_ phosphopeptide enrichment.

### Effect of kinase inhibitors on phosphorylation of CnaA *in vivo*

To determine the effect of GSK-3β and CK1 inhibitors on the phosphorylation status of CnaA *in vivo*, the CnaA-EGFP expression strain was grown in presence of 0.75 µM each of the GSK-3β inhibitor VII (calbiochem) and D4476 (abcam) for a period of 24 h and the isolated CnaA-EGFP fusion protein was subjected to phospho enrichment and mass spectrometry as described earlier [51].

### *Aspergillus fumigatus* calcineurin modeling

Homology models for the *A. fumigatus* calcineurin A and B subunits were prepared using the Phyre server. A model of the fungal heterodimer was constructed by superimposing the individually created homology models onto the X-ray structure of the human calcineurin heterodimer bound to a substrate peptide (PDB ID 2P6B) [38]. Superpositions and analysis of mutations were performed using PyMol, Coot and Molprobity. Due to the high level of sequence conservation with the mammalian calcineurin structure already determined, the models are largely superimposable at the Cα backbone level and exhibit good packing overall. We employed this model of *A. fumigatus* calcineurin to propose a structural basis for the disruption of calcineurin activity observed for the mutants described in the current study.

### Murine inhalational model of invasive aspergillosis

Six-week-old CD1 male mice (mean weight 22.5 g) were immunosuppressed with both cyclophosphamide and triamcinolone acetonide as previously described [52]. Two groups of 20 immunosuppressed, unanesthetized mice each inhaled an aerosolized suspension of either AF293 wild-type or CnaA^mt^-4SA strain [52]. Survival was plotted on a Kaplan-Meier curve and log rank was used for pair-wise comparison of survival with statistical significance defined as a two-tailed p<0.05. Histopathological examination of the lungs was performed in two mice in each group that were euthanized on day +7 of infection. Lungs were embedded in 10% neutral buffered formalin and subsequently sectioned and stained with Gomori methenamine silver and hematoxylin-eosin for assessment of histological signs of infection. The animal model and experiments were conducted in accordance with the Animal Care and Use Program of the Duke University Medical Center.

## Supporting Information

Figure S1 **Clustal alignment of the calcineurin A proteins from 24 different species of the region including the CnBBH and the CaMBD.** While there was clear homology observed in the CnBBH and the CaMBD, the linker that separates these conserved domains exhibited variation. Notably, filamentous fungal species contained several serine and proline residues in this linker. AfcnaA-*Aspergillus fumigatus* CnaA; AmCnaA-*Allomyces macrogynus*; BcCnaA-*Botrytis cinerea* CnaA; BchycnaA-*Batrachochytrium dendrobatidis* CnaA; CaCnaA-*Candida albicans* CnaA; CcCnaA-*Coprinopsis cinerea* CnaA; CeCnaA-*Caenorhabditis elegans* CnaA; CiCnaA-*Coccidioides immitis*; CnCnaA-*Cryptococcus neoformans* CnaA; DdCnaA-*Dictyostelium discoideum* CnaA; FvCnaA-*Fusarium verticillioides* CnaA; HcCnaA-*Histoplasma capsulatum* CnaA; HcnaA-*Homo sapiens* CnaA; MgCnaA-*Magnaporthe grisea* CnaA; MygCnaA-*Microsporum gypseum* CnaA; MvCnaA-*Mortierella verticillata* CnaA; NcCnaA-*Neurospora crassa* CnaA; PbCnaA-*Paracoccidiodes brasilensis* CnaA; PtCnaA- *Puccinia triticina* CnaA; RoCnaA-*Rhizopus oryzae* CnaA; ScCnaA-*Saccharomyces cerevisiae* CnaA; SpCnaA-*Schizosaccharomyces pombe* CnaA; TrCnaA-*Trichophyton rubrum* CnaA; UmCnaA-*Ustilago maydis* CnaA.
(PDF)

Figure S2 **Phosphorylation of the** ***A. fumigatus*** **CnaB regulatory subunit.** Annotated tandem mass spectrum of RA[pS]VGTSQLLDNIV[pS]ASNFDRDEVDR (3^+^ m/z 1008.7813) from CnaB revealed two unique phosphorylated serine residues (Ser21 and Ser33) localized with >99% confidence using AScore. The presence of each identified C-terminal (y) and N-terminal (b) product ions are indicated within the peptide sequence.
(PDF)

Figure S3 **Tandem mass spectra of phosphorylated peptides identified from** ***Magnaporthe grisea*** **calcineurin.** Phosphorylation in the SPRR (A) and the C-terminus (B) are shown. Annotated tandem mass spectra showing assigned fragment ion assignments from Mascot database searches imported into Scaffold are shown. For each qualitative identification, spectra were subjected to Ascore localization to assign percent confidences of amino acid specific assignments. An extracted ion chromatogram (EIC) of the precursor m/z (+/−20 ppm) of the chromatographic elution profile of each phosphorylated peptide is presented to the right of each annotated fragment ion spectra. An asterisk in the EIC plot indicated the peak which was chosen for subsequent qualitative identification.
(PDF)

Figure S4 **Tandem mass spectra of phosphorylated peptides identified from** ***Neurospora crassa*** **calcineurin.** Annotated tandem mass spectra showing assigned fragment ion assignments from Mascot database searches imported into Scaffold are shown. For each qualitative identification, spectra were subjected to Ascore localization to assign percent confidences of amino acid specific assignments. An extracted ion chromatogram (EIC) of the precursor m/z (+/−20 ppm) of the chromatographic elution profile of each phosphorylated peptide is presented to the right of each annotated fragment ion spectra. An asterisk in the EIC plot indicated the peak which was chosen for subsequent qualitative identification.
(PDF)

Figure S5 **Tandem mass spectra of phosphorylated peptides identified from** ***Mucor circinelloides*** **calcineurin.** Phosphorylation in the SPRR (A) and the C-terminus (B) are shown. Annotated tandem mass spectra showing assigned fragment ion assignments from Mascot database searches imported into Scaffold are shown. For each qualitative identification, spectra were subjected to Ascore localization to assign percent confidences of amino acid specific assignments. An extracted ion chromatogram (EIC) of the precursor m/z (+/−20 ppm) of the chromatographic elution profile of each phosphorylated peptide is presented to the right of each annotated fragment ion spectra. An asterisk in the EIC plot indicated the peak which was chosen for subsequent qualitative identification.
(PDF)

Figure S6 **Extracted ion chromatogram (EIC) peak height measurements of CnaA following TiO_2_ enrichment and LC-MS/MS analysis.** Manual EIC generation was performed for each precursor ion (+/−20 ppm tolerance) which was qualitatively identified as a phosphorylated peptide from CnaA and resulting peak height was used as a measure of intensity. To ensure the same precursor ion was being compared across the +FK506, −FK506, and Control samples, a peak was required to have the same m/z (+/−10 ppm), have the same retention time (+/−30 seconds), and was qualitatively identified as the same species in Mascot database searches.
(PDF)

Figure S7 **Extracted ion chromatogram (EIC) peak height measurements of CnaB following TiO_2_ enrichment and LC-MS/MS analysis.** Manual EIC generation was performed for each precursor ion (+/−20 ppm tolerance) which was qualitatively identified as a phosphorylated peptide from CnaB and resulting peak height was used as a measure of intensity. To ensure the same precursor ion was being compared across the +FK506, −FK506, and Control samples, a peak was required to have the same m/z (+/−10 ppm), have the same retention time (+/−30 seconds), and was qualitatively identified as the same species in Mascot database searches.
(PDF)

Figure S8 **CnaA mutation strains cultured on GMM agar in the presence of varying concentrations of the cell wall inhibitor caspofungin for 5 days.** The THL-PLS mutation (catalytic residues) and the V371D mutation (CnBBH residue) in CnaA completely abolish calcineurin-mediated paradoxical growth. The NIR-AAA (PxIxIT Binding Motif) and the RVF-AAA (CaMBD residues) mutations do show slight paradoxical growth at 2 µg/ml and 4 µg/ml of caspofungin.
(PDF)

Table S1 **Strains used in the present study.** All the strains listed were constructed in the Af293 derived isogenic Af293.1 strain with *pyrG* auxotrophy. The Δ*cnaA* strain previously constructed in the Af293.1 background by utilizing the *pyrG* marker gene was transformed with the various *cnaA* constructs either under the control of its native promoter or the *otef* promoter with *gfp* tag and hygromycin (*hph*) resistance marker gene.
(DOCX)

Table S2 **Primers and constructs.** All the primers used for construction of the various *cnaA*.
(DOCX)
