# Supplementary material for: Phosphorylation of Calcineurin at a Novel Serine-Proline Rich Region Orchestrates Hyphal Growth and Virulence in Aspergillus fumigatus
Source: PLoS Pathog. 2013 Aug 22;9(8):e1003564. doi: 10.1371/journal.ppat.1003564 (PMC3749960; doi:10.1371/journal.ppat.1003564)
Supplement: Figure S1 — Clustal alignment of the calcineurin A proteins from 24 different species of the region including the CnBBH and the CaMBD. While there was clear homology observed in the CnBBH and the CaMBD, the linker that separates these conserved domains exhibited variation. Notably, filamentous fungal species contained several serine and proline residues in this linker. AfcnaA-Aspergillus fumigatus CnaA; AmCnaA-Allomyces macrogynus; BcCnaA-Botrytis cinerea CnaA; BchycnaA-Batrachochytrium dendrobatidis CnaA; CaCnaA-Candida albicans CnaA; CcCnaA-Coprinopsis cinerea CnaA; CeCnaA-Caenorhabditis elegans CnaA; CiCnaA-Coccidioides immitis; CnCnaA-Cryptococcus neoformans CnaA; DdCnaA-Dictyostelium discoideum CnaA; FvCnaA-Fusarium verticillioides CnaA; HcCnaA-Histoplasma capsulatum CnaA; HcnaA-Homo sapiens CnaA; MgCnaA-Magnaporthe grisea CnaA; MygCnaA-Microsporum gypseum CnaA; MvCnaA-Mortierella verticillata CnaA; NcCnaA-Neurospora crassa CnaA; PbCnaA-Paracoccidiodes brasilensis CnaA; PtCnaA- Puccinia triticina CnaA; RoCnaA-Rhizopus oryzae CnaA; ScCnaA-Saccharomyces cerevisiae CnaA; SpCnaA-Schizosaccharomyces pombe CnaA; TrCnaA-Trichophyton rubrum CnaA; UmCnaA-Ustilago maydis CnaA. (PDF) [file ppat.1003564.s001.pdf]

CnBBH | Linker (includes the SPRR) | CaMBD

```
Majority NNVMNIRQFNCTPHPYWLPNFMDVFTWSLPFVGEKITDMLIAILNTCSKEELE-E------EDE-----SXSP----PXS
                  10        20        30        40        50        60        70        80

AfcnaA   NNVMNIRQFNCTPHPYWLPNFMDVFTWSLPFVGEKITDMLIAILNTCSKEELE-D------ETPT----SVSPS---APS 66
AmCnaA   NNVMNIRQFNCTPHPYWLPNFMDVFTWSLPFVGEKITDMLVAVLNVCSKEELD-E------GRE---------------- 57
BcCnaA   NNVMNIRQFNCTPHPYWLPNFMDVFTWSLPFVGEKITDMLLAILSTSSEDELKDD------QTPS----STSPG---PIS 67
BdCnaA   NNVMNIRQFNCSPHPYWLPNFMDVFTWSLPFVGEKITDMLLAILNICSKEELA-D------EDEI----QHVEG------ 63
CacnaA   NNVMNIRQFNASPHPYWLPHFMDVFTWSLPFVGEKVTDMLVSILNVCTEEELDEDLP--FSEAEIGVTPTTTTTGATPVS 78
CcCnaA   -NVMNIRQFNCTPHPYWLPNFMDVFTWSLPFVGEKITDMLVAVLNTCTKEELE-E------PDED----LALIS---PT- 64
CeCnaA   NNVMNIRQFNCSPHPYWLPNFMDVFTWSLPFVGEKVTEMLVHILNICSDDELMAEC-----DDT---------------- 59
CicnaA   NNVMNIRQFNCTPHPYWLPNFMDVFTWSLPFVGEKITDMLIAILNTCSKEELE-E------EPS-----AFSSA---PAS 65
CnCnaA   -NVMNIRQFNCTPHPYWLPNFMDVFTWSLPFVGEKITDMLIAILNCCTKEELE-E------EDEE----FPLNA---PEP 65
DdCnaA   NNVMNIRQFTCSPHPYWLPNFMDVFTWSMPFVSEKVAEMLLVLLNLCNDEEAE--------KNEN----AQTVK------ 62
FvCnaA   NNVMNIRQFNCTPHPYWLPNFMDVFTWSLPFVGEKITDMLIAILSTCSEEELK-E------ETPS----STSPG---PAS 66
HcCnaA   NNVMNIRQFNCTPHPYWLPNFMDVFTWSLPFVGEKITDMLIAILNTCSKEELE-E------EPSS----SVSSD---RAS 66
HcnaA    NNVMNIRQFNCSPHPYWLPNFMDVFTWSLPFVGEKVTEMLVNVLNICSDDELGSE------EDG---------------- 58
MccnaA   NNVMNIRQFNCTPHPYWLPNFMDVFSWSLPFVGEKITDMLIAVLNVCSKEELAED------DKQL----IEDK----SN- 65
MccnaB   DNVMNIRQFNSTPHPYWLPNFMNVVDWSLPFVGEKVTNMLLAILSICTKEELLAE------DDQA----HHEK----END 66
MccnaC   KSVLNIRQFNSSPHPYWLPNFMNVFNWSLPFVGEKITSMLLALLNICSQEELAAT------PEQK----AMESPVVVVPE 70
MgCnaA   NNVMNIRQFNCTPHPYWLPNFMDVFTWSLPFVGEKITDMLLAILGTCSEEELR-E------GDSS----STSPG---PQS 66
MgyCnaA  NNVMNIRQFNCSPHPYWLPNFMDVFTWSLPFVGEKITDMLIAILNTCSKEELE-E------ETPL----SASET---PPS 66
MvCnaA   NNVMNIRQFNATPHPYWLPNFMDVFTWSLPFVGEKITDMLLAILNTCSKEELA-E------DDMI----LNLTD---GTS 66
NcCnaA   NNVMNIRQFNCTPHPYWLPNFMDVFTWSLPFVGEKITDMLIAILSTCSEEELR--------EDSA----TTSPG---SAS 65
PbcnaA   NNVMNIRQFNCTPHPYWLPNFMDVFTWSLPFVGEKITDMLIAILNTCSKEELE-E------ESP-----SVSG----AAS 64
PtCnaA   NNVMNIRQFNCTPHPYWLPNFMDVFTWSLPFVGEKITDMLIAILGICTKEELD-E------EEEE----LPLAS---EEV 66
RoCnaA   NNVMNIRQFNCTPHPYWLPNFMDVFTWSLPFVGEKITDMLISVLNVCSKEELAED------D-QL----LEEK----P-- 63
ScCnaA   ENVMNIRQFHMSPHPYWLPDFMDVFTWSLPFVGEKVTSMLVSILNICSEQELDPESEPKAAEETVKARANATKETGTPSD 80
SpCnaA   NNVMNIRQFNCSPHPYWLPNFMDVFTWSLPFVGEKVSEMLISMLNICSKEELYET------DLKE----SAPTQHKQPAP 70
TrCnaA   NNVMNIRQFNCSPHPYWLPNFMDVFTWSLPFVGEKITDMLIAILNTCSKEELE-E------ETPL----SASEA---PPS 66
UmCnaA   NNVMNIRQFNCTPHPYWLPNFMDVFTWSLPFVGEKITDMLIAILNVCSKEELE-E------EEEE----DEIPT---TPT 66
```

```
Majority P-------------------------------MDPESX-EFRRRAIKNKILAIGRMSRVFQVLREESE
                  90        100       110       120       130       140       150

AfcnaA   P---------PLP-------------------MDVESS-EFKRRAIKNKILAIGRLSRVFQVLREESE 105
AmCnaA   --------------------------------HVLDDE----RRQVIKNKILAVGRMSKMFNLLRQEAE 90
BcCnaA   P---------PIN-----------------SPMDPESI-EYKRRAIKNKILAIGRLSRVFQVLREESE 108
BdCnaA   --------------------------------EELTEQ-EQRRVVIRNKIKAVGRMARVFATLREEKE 98
CacnaA   PKAYPPSSRITSPHKSTKLVESRANEEEKANDGDDDSEMTLEEKKQALRNKIIAIGKMSRMFQVLREEQE 148
CcCnaA   --------------------------------AAAEES-AERRKIIKNKIMAVGRMARVFALLREESE 99
CeCnaA   --------------------------------FEGGVG-SARKEVIRHKIRAIGKMARAFSVLRHESE 94
CicnaA   P---------PLP-------------------MDTDSA-EFKRRAIKNKILAIGRLSRVFQVLREESE 104
CnCnaA   --------------------------------TDAESA-AERRQIIKNKILAVGRMSRVFSLLREESE 100
DdCnaA   --------------------------------DTSEE--EKRRQMLRAKVKSVSKMMRMFSLLRQERE 96
FvCnaA   P---------ALS-----------------N--DPESI-EVRRRAIKNKILAIGRLSRVFQVLREESE 105
HcCnaA   P---------PLP-------------------MDPEST-EFKRRAIKNKILAIGRLSRVFQVLREEAE 105
HcnaA    --------------------------------FDGATA-AARKEVIRNKIRAIGKMARVFSVLREESE 93
MccnaA   --------------------------------NLDDA-EQRRIVIRNKIMAVGKISRVFSVLRENSE 99
MccnaB   Q-------------------------------QELDEI-EQRKIVIRNKILAIGKISRVYSVLRENSE 102
MccnaC   N-------------------------------ADMTP--EQRRQIIRNKIMAIGKMSRTFSVLRENSE 105
MgCnaA   P---------PQTPALPEAAGDGEGSSTSANAAAMDPESI-EFKRRAIKNKILAIGRLSRVFQVLREESE 126
MgyCnaA  P---------TLPS------------------NDPEST-EFKRRAIKNKILAIGRLSRVFQVLREESE 106
MvCnaA   AVSAMEVDTAPISSGSSHTSVS-TVSSIQDEDMTDAMTER-EANKNRIRQKILAIGRISRVFAVLRNESE 134
NcCnaA   P---------ALP----------------SAANQDPDSI-EFKRRAIKNKILAIGRLSRVFQVLREESE 108
PbcnaA   P---------ALQ-------------------MDPEST-EFKRRAIKNKILAIGRLSRVFQVLREESE 103
PtCnaA   E---------SDPD-----------------SPSAAAS-AERRQAIKNKIMAVGKMSRVFAVLREEAE 107
RoCnaA   --------------------------------VTDDA-EQRRIVIRNKIMAVGKMSRVFSVLRENSE 97
ScCnaA   E------------------------------KASSAILEDETRRKALRNKILAIAKVSRMFSVLREESE 119
SpCnaA   S------------------------ENENKADQEIDI-EARRQIIKNKIMAIGRISRVFSVLREERE 112
TrCnaA   P---------TLPS------------------TDPEST-EFKRRAIKNKILAIGRLSRVFQVLREESE 106
UmCnaA   --------------------------------SAAEEET-AERRTLIKNKILAVGRMSRVFALLREEAE 102
```
