# Supplementary figures and images for: Phosphorylation of Calcineurin at a Novel Serine-Proline Rich Region Orchestrates Hyphal Growth and Virulence in Aspergillus fumigatus
Source: PLoS Pathog. 2013 Aug 22;9(8):e1003564. doi: 10.1371/journal.ppat.1003564 (PMC3749960; doi:10.1371/journal.ppat.1003564)

<sup>PO<sub>4</sub></sup>  
 19-RASVGTSQLLDNIVSASNFRDEVDR-44  
<sup>PO<sub>4</sub></sup>

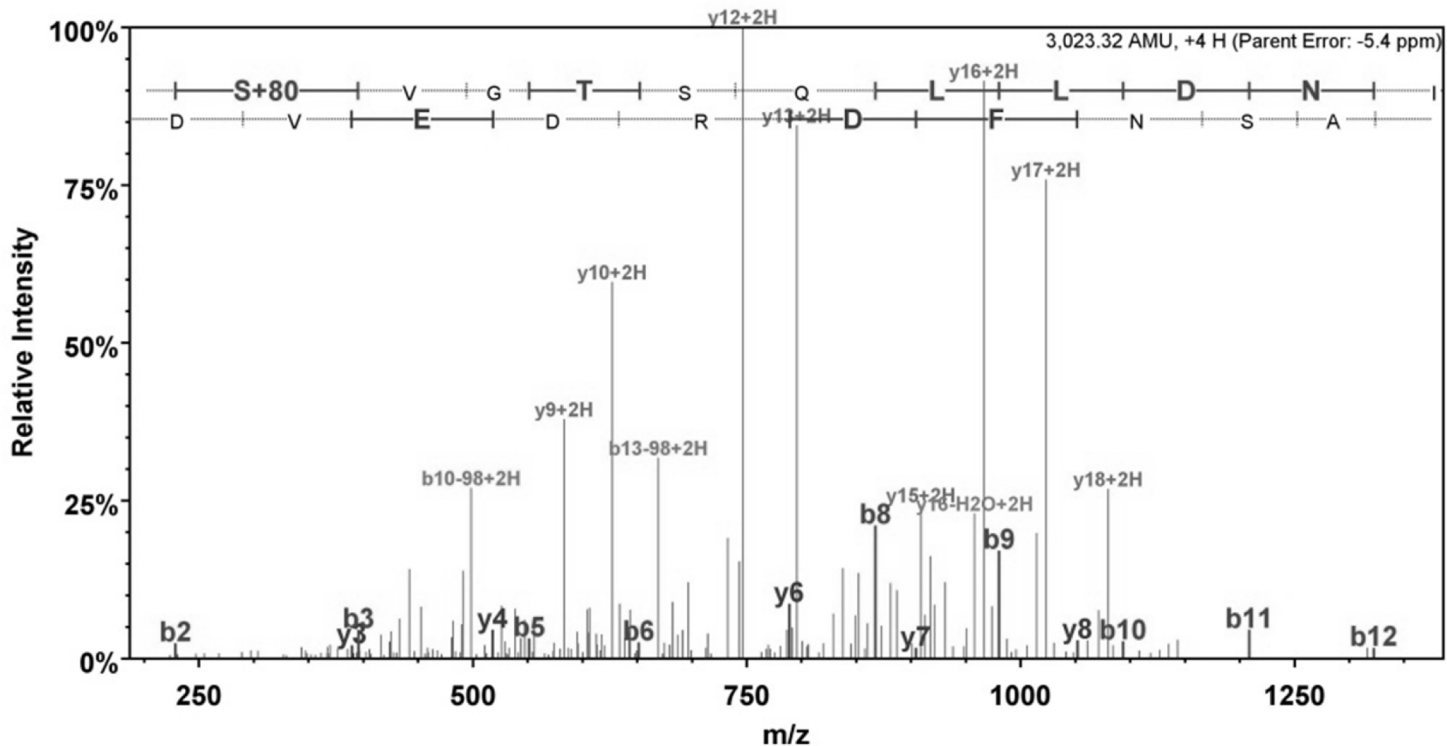

Supplement: Figure S2 — Phosphorylation of the A. fumigatus CnaB regulatory subunit. Annotated tandem mass spectrum of RA[pS]VGTSQLLDNIV[pS]ASNFDRDEVDR (3+ m/z 1008.7813) from CnaB revealed two unique phosphorylated serine residues (Ser21 and Ser33) localized with >99% confidence using AScore. The presence of each identified C-terminal (y) and N-terminal (b) product ions are indicated within the peptide sequence. (PDF) [file ppat.1003564.s002.pdf]

# 417- EDSATTSPGSASPALPSAANQDPDSIEFK -445

Mascot Ion Score: 36.1

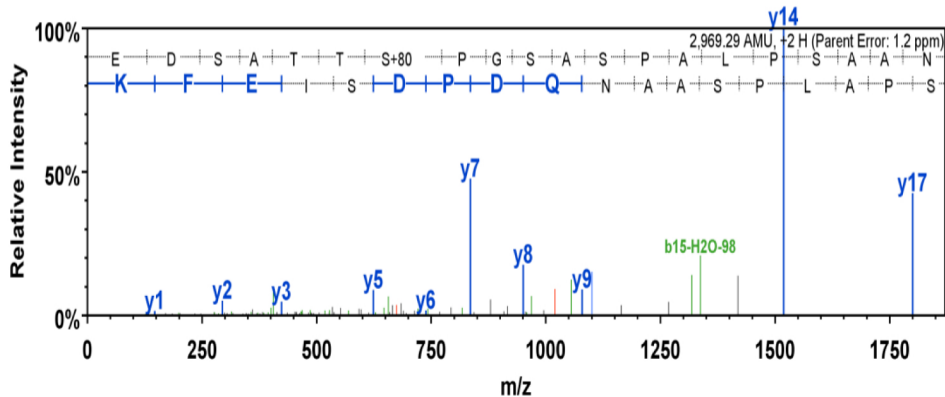

## Extracted Ion Chromatogram 1485.65 m/z (20 ppm)

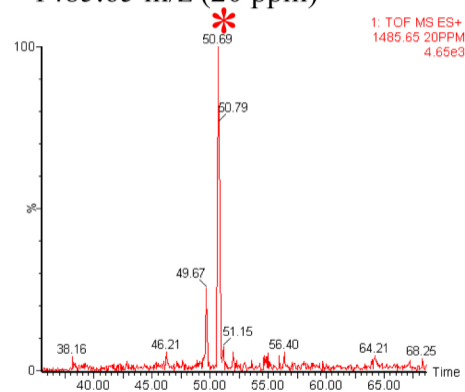

Supplement: Figure S4 — Tandem mass spectra of phosphorylated peptides identified from Neurospora crassa calcineurin. Annotated tandem mass spectra showing assigned fragment ion assignments from Mascot database searches imported into Scaffold are shown. For each qualitative identification, spectra were subjected to Ascore localization to assign percent confidences of amino acid specific assignments. An extracted ion chromatogram (EIC) of the precursor m/z (+/−20 ppm) of the chromatographic elution profile of each phosphorylated peptide is presented to the right of each annotated fragment ion spectra. An asterisk in the EIC plot indicated the peak which was chosen for subsequent qualitative identification. (PDF) [file ppat.1003564.s004.pdf]

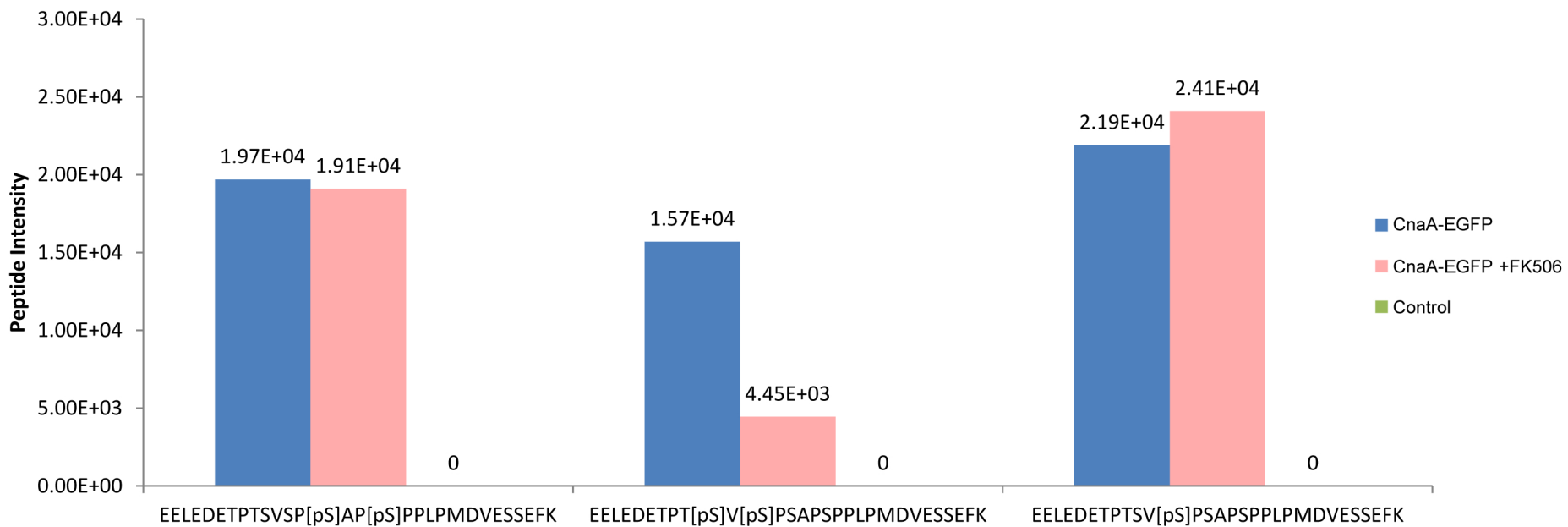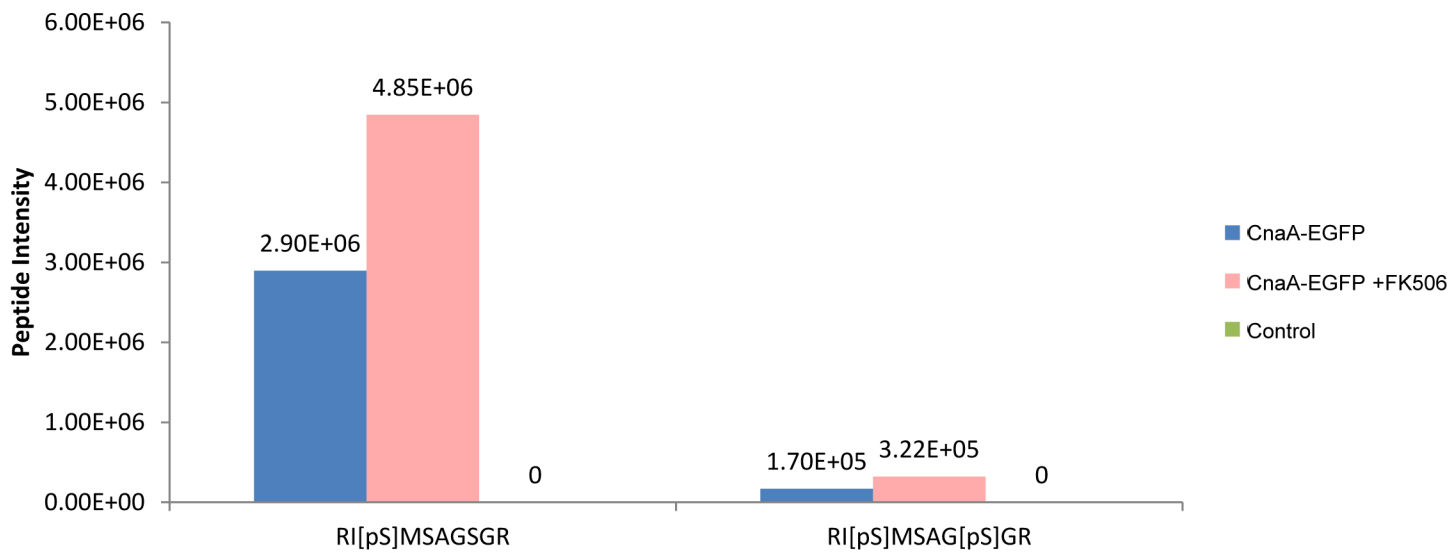

Supplement: Figure S6 — Extracted ion chromatogram (EIC) peak height measurements of CnaA following TiO2 enrichment and LC-MS/MS analysis. Manual EIC generation was performed for each precursor ion (+/−20 ppm tolerance) which was qualitatively identified as a phosphorylated peptide from CnaA and resulting peak height was used as a measure of intensity. To ensure the same precursor ion was being compared across the +FK506, −FK506, and Control samples, a peak was required to have the same m/z (+/−10 ppm), have the same retention time (+/−30 seconds), and was qualitatively identified as the same species in Mascot database searches. (PDF) [file ppat.1003564.s006.pdf]

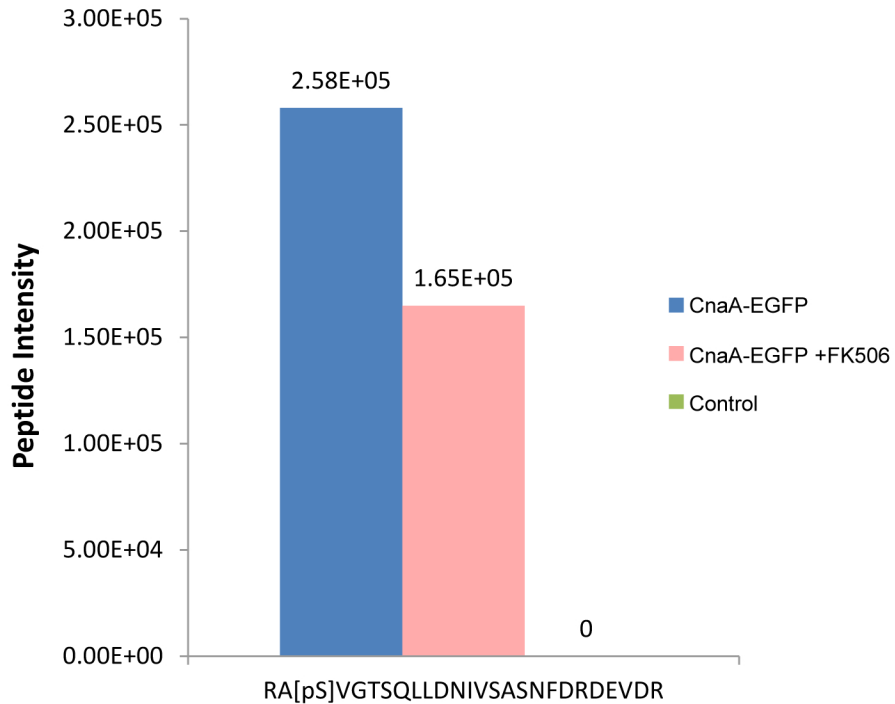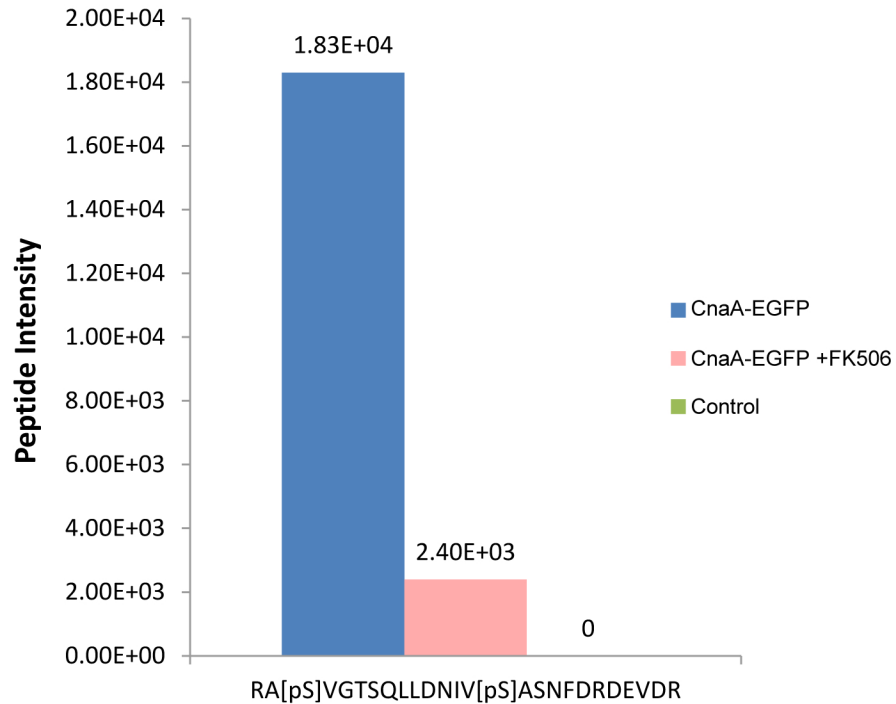

Supplement: Figure S7 — Extracted ion chromatogram (EIC) peak height measurements of CnaB following TiO2 enrichment and LC-MS/MS analysis. Manual EIC generation was performed for each precursor ion (+/−20 ppm tolerance) which was qualitatively identified as a phosphorylated peptide from CnaB and resulting peak height was used as a measure of intensity. To ensure the same precursor ion was being compared across the +FK506, −FK506, and Control samples, a peak was required to have the same m/z (+/−10 ppm), have the same retention time (+/−30 seconds), and was qualitatively identified as the same species in Mascot database searches. (PDF) [file ppat.1003564.s007.pdf]

Caspofungin ( $\mu\text{g/ml}$ )

0

1

2

4

WT

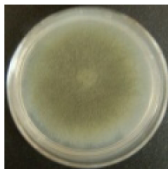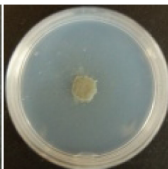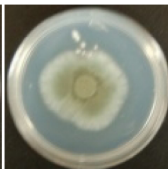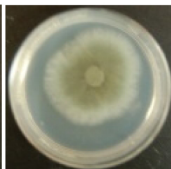

NIR-AAA

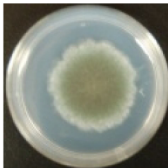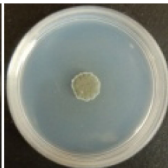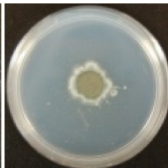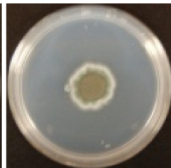

RVF-AAA

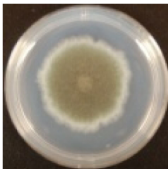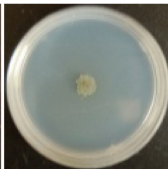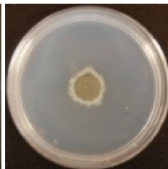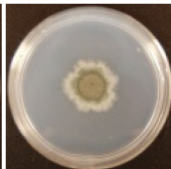

V371D

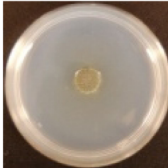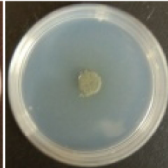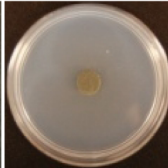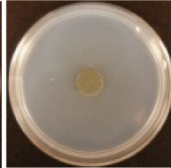

THL-PLS

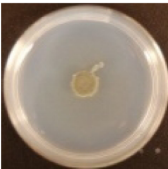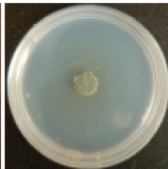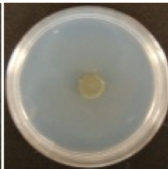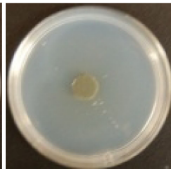

Supplement: Figure S8 — CnaA mutation strains cultured on GMM agar in the presence of varying concentrations of the cell wall inhibitor caspofungin for 5 days. The THL-PLS mutation (catalytic residues) and the V371D mutation (CnBBH residue) in CnaA completely abolish calcineurin-mediated paradoxical growth. The NIR-AAA (PxIxIT Binding Motif) and the RVF-AAA (CaMBD residues) mutations do show slight paradoxical growth at 2 µg/ml and 4 µg/ml of caspofungin. (PDF) [file ppat.1003564.s008.pdf]
