# Supplementary material for: Phosphorylation of Calcineurin at a Novel Serine-Proline Rich Region Orchestrates Hyphal Growth and Virulence in Aspergillus fumigatus
Source: PLoS Pathog. 2013 Aug 22;9(8):e1003564. doi: 10.1371/journal.ppat.1003564 (PMC3749960; doi:10.1371/journal.ppat.1003564)

A **420-EGDSSSTSPGPQS*PPQT*PALPEAAGDGEGSSTSANAAAMDPESIEFK-466**

Mascot Ion Score: 56.0

Ascore Localization: <50%

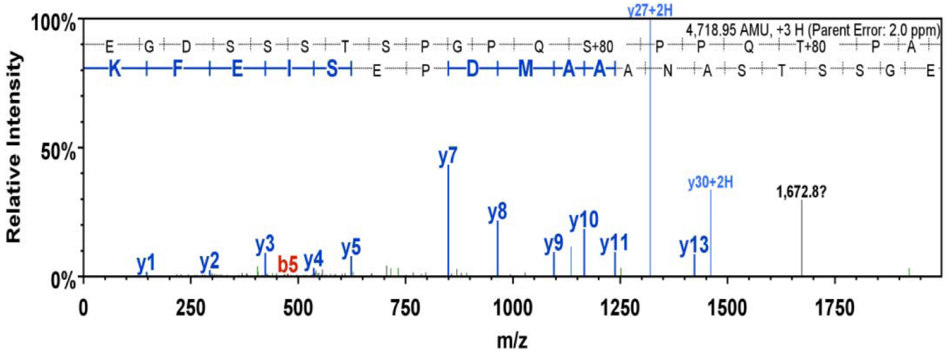

Extracted Ion Chromatogram

1574.28 m/z (20 ppm)

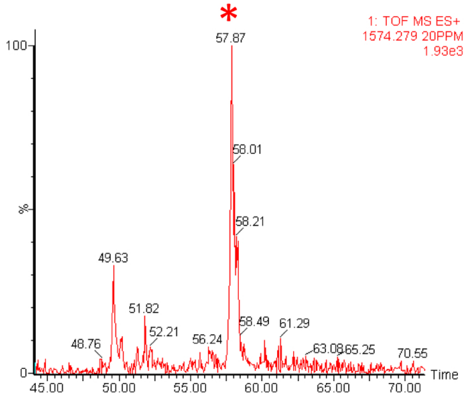

B **575-RLS*TSSSSDR-584**

Mascot Ion Score: 44.2

Ascore Localization: <80%

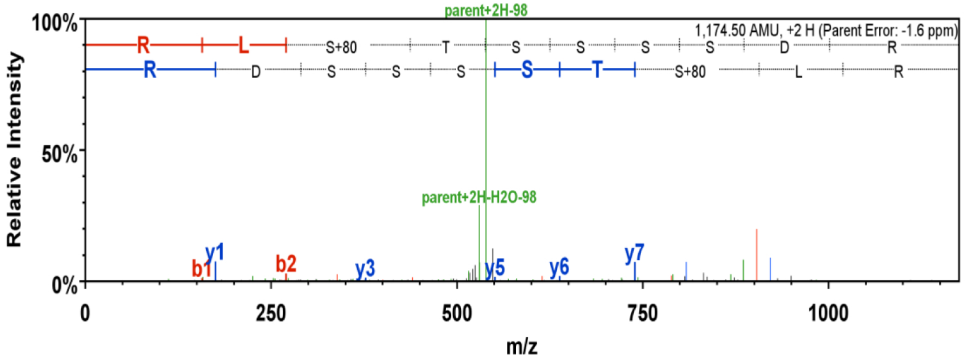

Extracted Ion Chromatogram

588.26 m/z (20 ppm)

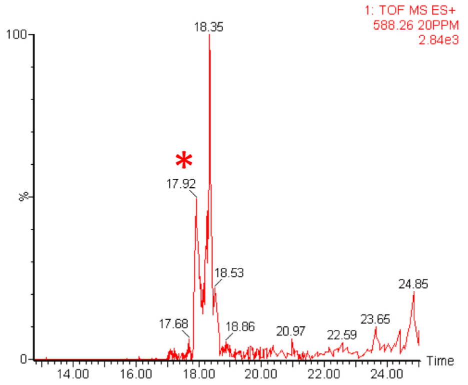

Supplement: Figure S3 — Tandem mass spectra of phosphorylated peptides identified from Magnaporthe grisea calcineurin. Phosphorylation in the SPRR (A) and the C-terminus (B) are shown. Annotated tandem mass spectra showing assigned fragment ion assignments from Mascot database searches imported into Scaffold are shown. For each qualitative identification, spectra were subjected to Ascore localization to assign percent confidences of amino acid specific assignments. An extracted ion chromatogram (EIC) of the precursor m/z (+/−20 ppm) of the chromatographic elution profile of each phosphorylated peptide is presented to the right of each annotated fragment ion spectra. An asterisk in the EIC plot indicated the peak which was chosen for subsequent qualitative identification. (PDF) [file ppat.1003564.s003.pdf]
