# Supplementary material for: Phosphorylation of Calcineurin at a Novel Serine-Proline Rich Region Orchestrates Hyphal Growth and Virulence in Aspergillus fumigatus
Source: PLoS Pathog. 2013 Aug 22;9(8):e1003564. doi: 10.1371/journal.ppat.1003564 (PMC3749960; doi:10.1371/journal.ppat.1003564)
Supplement: Figure S5 — Tandem mass spectra of phosphorylated peptides identified from Mucor circinelloides calcineurin. Phosphorylation in the SPRR (A) and the C-terminus (B) are shown. Annotated tandem mass spectra showing assigned fragment ion assignments from Mascot database searches imported into Scaffold are shown. For each qualitative identification, spectra were subjected to Ascore localization to assign percent confidences of amino acid specific assignments. An extracted ion chromatogram (EIC) of the precursor m/z (+/−20 ppm) of the chromatographic elution profile of each phosphorylated peptide is presented to the right of each annotated fragment ion spectra. An asterisk in the EIC plot indicated the peak which was chosen for subsequent qualitative identification. (PDF) [file ppat.1003564.s005.pdf]

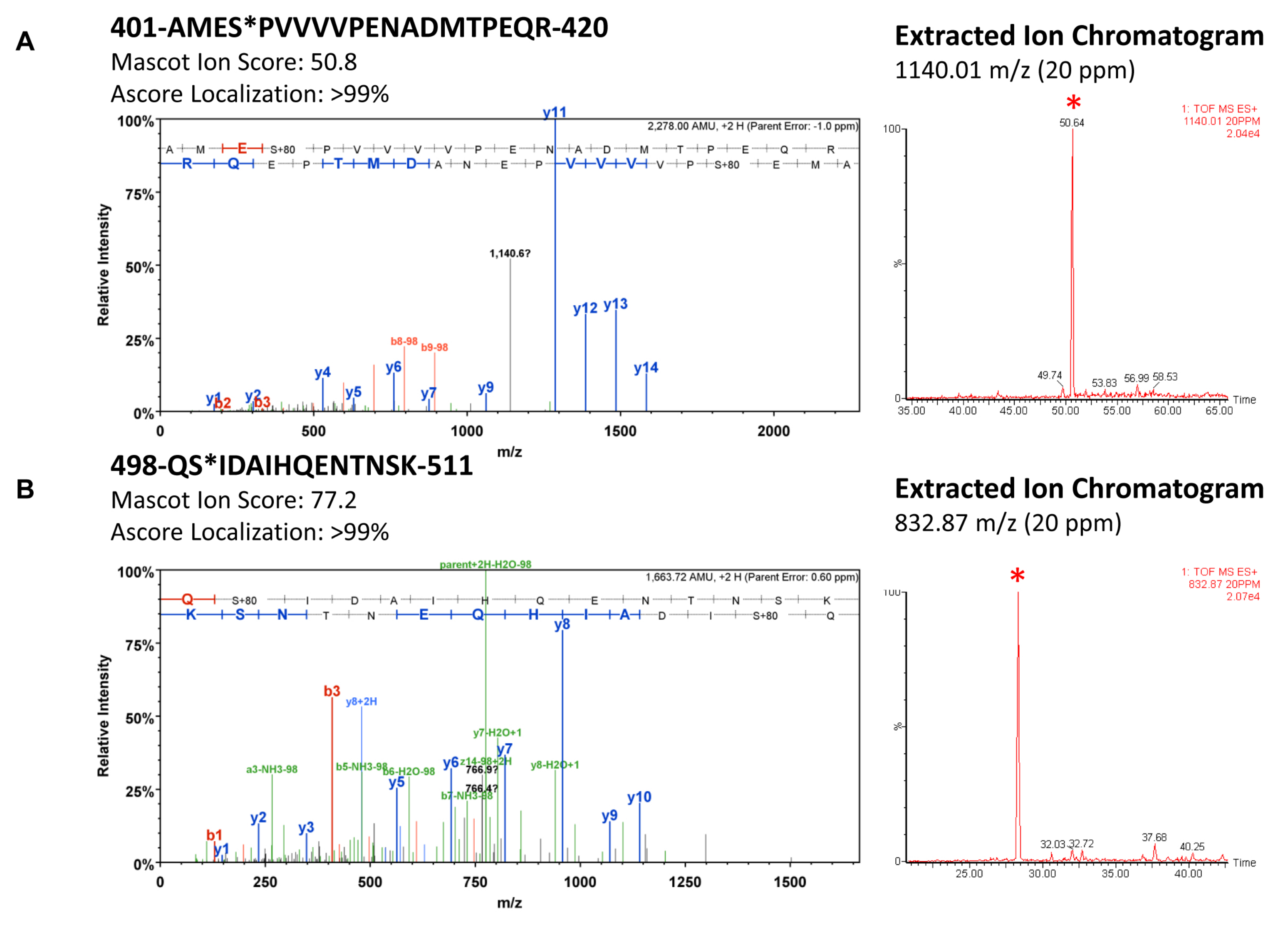
A
401-AMES*PVVVVPENADMTPEQR-420
Mascot Ion Score: 50.8
Ascore Localization: >99%
2,278.00 AMU, +2 H (Parent Error: -1.0 ppm)
Relative Intensity
m/z
y11
1,140.6?
y12
y13
y14
b8-98
b9-98
y4
y6
y5
y7
y9
y1
b2
y2
b3
Extracted Ion Chromatogram
1140.01 m/z (20 ppm)
1: TOF MS ES+
1140.01 20PPM
2.04e4
50.64
49.74
53.83
56.99
58.53
Time
B
498-QS*IDAIHQENTNSK-511
Mascot Ion Score: 77.2
Ascore Localization: >99%
1,663.72 AMU, +2 H (Parent Error: 0.60 ppm)
parent+2H-H2O-98
Relative Intensity
m/z
y8
b3
y8+2H
y7-H2O+1
y7
y6
z14-98+2H
766.9?
766.4?
y8-H2O+1
a3-NH3-98
b5-NH3-98
b6-H2O-98
b7-NH3-98
y5
y10
y9
y2
y3
b1
y1
Extracted Ion Chromatogram
832.87 m/z (20 ppm)
1: TOF MS ES+
832.87 20PPM
2.07e4
32.03
32.72
37.68
40.25
Time
