# Supplementary material for: Phosphorylation of Calcineurin at a Novel Serine-Proline Rich Region Orchestrates Hyphal Growth and Virulence in Aspergillus fumigatus
Source: PLoS Pathog. 2013 Aug 22;9(8):e1003564. doi: 10.1371/journal.ppat.1003564 (PMC3749960; doi:10.1371/journal.ppat.1003564)
Supplement: Table S1 — Strains used in the present study. All the strains listed were constructed in the Af293 derived isogenic Af293.1 strain with pyrG auxotrophy. The ΔcnaA strain previously constructed in the Af293.1 background by utilizing the pyrG marker gene was transformed with the various cnaA constructs either under the control of its native promoter or the otef promoter with gfp tag and hygromycin (hph) resistance marker gene. (DOCX) [file ppat.1003564.s009.docx]

**Table S1: Strains Used in the Present Study**

| **Strain** | **Parent Strain** | **Genotype** |
| --- | --- | --- |
| Af293 |  | Wild type |
| Δ*cnaA* | Af293.1 | Δ*cnaA*::*pyrG1* |
| AKO-AfCnaA | Af293.1 | Δ*cnaA*::*pyrG1* *cnaApromo***-***cnaA***-***gfp***-***hph* |
| AKO-CnaA**-**T1 | Af293.1 | Δ*cnaA*::*pyrG1* *cnaApromo***-***cnaAt1***-***gfp***-***hph* |
| AKO-CnaA**-**T2 | Af293.1 | Δ*cnaA*::*pyrG1* *cnaApromo***-***cnaAt2***-***gfp***-***hph* |
| AKO-CnaA-T3 | Af293.1 | Δ*cnaA*::*pyrG1* *cnaApromo***-***cnaAt3***-***gfp***-***hph* |
| AKO-CnaA-T4 | Af293.1 | Δ*cnaA*::*pyrG1* *cnaApromo***-***cnaAt4***-***gfp***-***hph* |
| OCNAG3 | Af293.1 | Δ*cnaA*::*pyrG1* *otefpromo***-***cnaA***-***gfp***-***hph* |
| AKO-CnCNA1 | Af293.1 | Δ*cnaA*::*pyrG1* *otefpromo***-***cncna1***-***gfp***-***hph* |
| AKO-CNAFCNA | Af293.1 | Δ*cnaA*::*pyrG1* *otefpromo***-***cnafcna***-***gfp***-***hph* |
| AKO-MccnaA | Af293.1 | Δ*cnaA*::*pyrG1* *otefpromo***-***mccnaA***-***gfp***-***hph* |
| AKO-MccnaC | Af293.1 | Δ*cnaA*::*pyrG1* *otefpromo***-***mccnaC***-***gfp***-***hph* |
| AKO-MgcnaA | Af293.1 | Δ*cnaA*::*pyrG1* *otefpromo***-***mgcnaA***-***gfp***-***hph* |
| AKO-NccnaA | Af293.1 | Δ*cnaA*::*pyrG1* *otefpromo***-***nccnaA***-***gfp***-***hph* |
| AKO-cnaA^mt^-4SA | Af293.1 | Δ*cnaA*::*pyrG1* *cnaApromo***-***cnaA^mt^***-***4SA***-***gfp***-***hph* |
| AKO-cnaA^mt^-4SE | Af293.1 | Δ*cnaA*::*pyrG1* *cnaApromo***-***cnaA^mt^***-***4SE***-***gfp***-***hph* |
| AKO-cnaA^mt^-NIR-AAA | Af293.1 | Δ*cnaA*::*pyrG1* *cnaApromo***-***cnaA^mt^***-***NIR***-***AAA***-***gfp***-***hph* |
| AKO-cnaA^mt^-THL-PLS | Af293.1 | Δ*cnaA*::*pyrG1* *cnaApromo***-***cnaA^mt^***-***THL***-***PLS***-***gfp***-***hph* |
| AKO-cnaA^mt^-V371D | Af293.1 | Δ*cnaA*::*pyrG1* *cnaApromo***-***cnaA^mt^***-***V371D***-***gfp***-***hph* |
| AKO-cnaA^mt^-RVF-AAA | Af293.1 | Δ*cnaA*::*pyrG1* *cnaApromo***-***cnaA^mt^***-***RVF-AAA***-***gfp***-***hph* |

All the strains listed were constructed in the Af293 derived isogenic Af293.1 strain with *pyrG* auxotrophy. The Δ*cnaA* strain previously constructed in the Af293.1 background by utilizing the *pyrG* marker gene was transformed with the various *cnaA* constructs either under the control of its native promoter or the *otef* promoter with *gfp* tag and hygromycin (*hph*) resistance marker gene.
