# Supplementary material for: Phosphorylation of Calcineurin at a Novel Serine-Proline Rich Region Orchestrates Hyphal Growth and Virulence in Aspergillus fumigatus
Source: PLoS Pathog. 2013 Aug 22;9(8):e1003564. doi: 10.1371/journal.ppat.1003564 (PMC3749960; doi:10.1371/journal.ppat.1003564)
Supplement: Table S2 — Primers and constructs. All the primers used for construction of the various cnaA. (DOCX) [file ppat.1003564.s010.docx]

**Table S2: Primers and constructs**

| **PRIMER** | **SEQUENCE (5'-3')** | **Direction** |
| --- | --- | --- |
| **Truncations of cnaA** |  |  |
| ***CnaA*** |  |  |
| GCNA-F2 | CGACGGATCCATGGATCAAGCACTGGCG | Forward |
| GCNA-R-Bam | CGACGGATCCGGCTTCCCTAGTCTC | Reverse |
| ***CnaA-T1*** |  |  |
| GCNA-F2 | CGACGGATCCATGGATCAAGCACTGGCG | Forward |
| VLKYE-R | CGACGGATCCCTCGTACTTCAGCAC | Reverse |
| ***CnaA-T2*** |  |  |
| GCNA-F2 | CGACGGATCCATGGATCAAGCACTGGCG | Forward |
| KEELE-R | CGACGGATCCTTCGAGCTCCTCCTTG | Reverse |
| ***CnaA-T3*** |  |  |
| GCNA-F2 | CGACGGATCCATGGATCAAGCACTGGCG | Forward |
| SSEF-R | CGACGGATCCGAATTCGCTGCTCTCCACG | Reverse |
| ***CnaA-T4*** |  |  |
| GCNA-F2 | CGACGGATCCATGGATCAAGCACTGGCG | Forward |
| VTELK-R | CGACGGATCCCTTGAGTTCCGTAAC | Reverse |
| **Cloning of Cryptococcus neoformans *CnCNA1*** | |  |
| Cn-cnaA-F | CATAGGATCCATGGCTTCCCCAGCCACTCAG |  |
| Cn-cnaA-R | CATAGGATCCCTCTCTCTCGCCTTGACCGCC |  |
| **Cloning of CnCNA1+AfcnaAchimera *CNAFCNA*** | |  |
| Cn-cnaA-F | CATAGGATCCATGGCTTCCCCAGCCACTCAG | Forward |
| CN-BBH-R | TGTCGGATGTTCATGACGTTTGATTCGTAC | Reverse |
| AF-BBH-F | AACGTCATGAACATCCGACAGTTCAACTGC | Forward |
| GCNA-R-Bam | CGACGGATCCGGCTTCCCTAGTCTC | Reverse |
| **Cloning of Mucor circinelloides *MccnaA*** | |  |
| MC-CnaA-F | CATAGGATCCATGGATGAGATTATTAGCCCA | Forward |
| MC-CnaA-R | CATAGGATCCCTGAAACATGGCATGAACAAC | Reverse |
| **Cloning of Mucor circinelloides *MccnaC*** | |  |
| MC-CnaC-F | CATAGGATCCATGTCTGATATAGCCATTACA | Forward |
| MC-CnaC-R | CATAGGATCCGACTTGAGGTTGGGCTCTACG | Reverse |
| **Cloning of Magnaporthe grisea *MgcnaA*** | |  |
| MgCnaA-F | ACGGGATCCCCCGGGATGGAGAATGACGGCGGCGCCAAT | Forward |
| MgCnaA-R | ACGGGATCCCCCGGGTTTCTTGCGGTCACTGCTAGACGA | Reverse |
| **Cloning of Neurospora crassa *NccnaA*** | |  |
| Nc-cnaA-F | CATAGGATCCATGGAAAGCAACAATGGTACC | Forward |
| Nc-cnaA-R | CATAGGATCCAGAAGTGCTGAGCCTCCTGGAG | Reverse |
| **Mutations of *cnaA*** |  |  |
| **cnaA^mt^-4SA mutation** |  |  |
| GCNA-F2 | CGACGGATCCATGGATCAAGCACTGGCG | Forward |
| cnaA-all4Ser-Ala-R | AGCAGCAGGTGCCACGGCGGTAGG | Reverse |
| cnaA-all4Ser-Ala-F | CCTACCGCCGTGGCACCTGCTGCT | Forward |
| GCNA-R-Bam | CGACGGATCCGGCTTCCCTAGTCTC | Reverse |
| **cnaA^mt^-4SE mutation** |  |  |
| GCNA-F2 | CGACGGATCCATGGATCAAGCACTGGCG | Forward |
| cnaA-4Ser-Glu-R | AGCGGAGGTTCCGGAGCTTCAGGTTCCACTTCGGTAGGAG | Reverse |
| cnaA-4Ser-Glu-F | CTCCTACCGAAGTGGAACCTGAAGCTCCGGAACCTCCGCT | Forward |
| GCNA-R-Bam | CGACGGATCCGGCTTCCCTAGTCTC | Reverse |
| **NIR-AAA** |  |  |
| GCNA-F2 | CGACGGATCCATGGATCAAGCACTGGCG | Forward |
| CNA-NIR-AAA-R | GTTGAACTGTGCGGCAGCCATGACGTTG | Reverse |
| CNA-NIR-AAA-F | CAACGTCATGGCTGCCGCACAGTTCAAC | Forward |
| GCNA-R-Bam | CGACGGATCCGGCTTCCCTAGTCTC | Reverse |
| **THL-PLS** |  |  |
| GCNA-F2 | CGACGGATCCATGGATCAAGCACTGGCG | Forward |
| CNA-THL-PLS-R | TGGCGACCAGTACGGGAGAGGCGGGCAG | Reverse |
| CNA-THL-PLS-F | CTGCCCGCCTCTCCCGTACTGGTCGCCA | Forward |
| GCNA-R-Bam | CGACGGATCCGGCTTCCCTAGTCTC | Reverse |
| **V371D** |  |  |
| GCNA-F2 | CGACGGATCCATGGATCAAGCACTGGCG | Forward |
| CNA-V371D-R | GCGACCACGTAAAGTCGTCCATGAAG | Reverse |
| CNA-V371D-F | CTTCATGGACGACTTTACGTGGTCGC | Forward |
| GCNA-R-Bam | CGACGGATCCGGCTTCCCTAGTCTC | Reverse |
| **RVF-AAA** |  |  |
| GCNA-F2 | CGACGGATCCATGGATCAAGCACTGGCG | Forward |
| CNA-RVF-AAA-R | CAACACCTGGGCGGCAGCCGAGAGACG | Reverse |
| CNA-RVF-AAA-F | CGTCTCTCGGCTGCCGCCCAGGTGTTG | Forward |
| GCNA-R-Bam | CGACGGATCCGGCTTCCCTAGTCTC | Reverse |

All the primers used for construction of the various *cnaA* constructs are listed.
